# Investigating the use of aquatic weeds as biopesticides towards promoting sustainable agriculture

**DOI:** 10.1371/journal.pone.0237258

**Published:** 2020-08-05

**Authors:** Yuting Fu, Jehangir H. Bhadha, Philippe Rott, Julien M. Beuzelin, Ramdas Kanissery

**Affiliations:** 1 Everglades Research and Education Center, Soil and Water Sciences Department, University of Florida, Belle Glade, Florida, United States of America; 2 Everglades Research and Education Center, Plant Pathology Department, University of Florida, Belle Glade, Florida, United States of America; 3 CIRAD, UMR BGPI, Montpellier, France; 4 BGPI, Univ Montpellier, CIRAD, INRAE, L’Institut Agro, Montpellier, France; 5 Everglades Research and Education Center, Entomology and Nematology Department, University of Florida, Belle Glade, Florida, United States of America; 6 Southwest Florida Research and Education Center, Horticultural Sciences Department, University of Florida, Immokalee, Florida, United States of America; Al-Azhar University, EGYPT

## Abstract

Aquatic weeds such as muskgrass (*Chara* spp.), water hyacinth (*Eichhornia crassipes*), water lettuce (*Pistia stratiotes*), hydrilla (*Hydrilla verticillate*), filamentous algae (*Lyngbya wollei*), and duckweed (*Lemna minor*) thrive in farm canals within the Everglades Agricultural Area of South Florida. Their presence, particularly during the summer months is an environmental concern with regards to water quality, in addition to being a nuisance because of their ability to multiply and spread rapidly in open waters causing restricted drainage/irrigation flow and low dissolved oxygen levels. Chemical control is effective but can have undesirable off-target effects, so reduced herbicide use is desirable. Hence, need exists to discover ways in which these weeds could be best managed or utilized. The objective of this research was to evaluate the allelopathic effect of these weeds to determine their use as potential biopesticides. Six aqueous extracts were tested against 100 bacterial strains isolated from plants and soil to evaluate their antimicrobial activity. These extracts were also used to determine their insecticidal and antifeedant effects on fall armyworm (FAW, *Spodoptera frugiperda*). Both extracts and powder form of the aquatic weeds were tested for their herbicidal activity towards seed germination and growth of three common terrestrial weed species. At a dilution of 1:100 and 1:1,000, none of the aquatic weeds inhibited *in-vitro* growth of the bacterial strains, with one exception (filamentous algae extract at 1:100 reduced growth of one bacterial isolate by 54%). Water lettuce reduced the survival rate of FAW by 14% while hydrilla and duckweed caused 11% and 9% reduction of FAW growth, respectively. Powdered duckweed inhibited the growth of nutsedge by 41%, whereas filamentous algae powder and extract reduced germination of amaranth by 20% and 28%, respectively. Harvesting these weeds and converting them into useable compounds could not only eliminate the in situ farm canal and water quality problems but also result in development of new soil amendments or biopesticides.

## Introduction

Aquatic weeds such as muskgrass (*Chara* spp., Charales: *Characeae*), water hyacinth (*Eichhornia crassipes*, Commelinales: *Pontederiaceae*), water lettuce (*Pistia stratiotes*, Alismatales: *Araceae*), hydrilla (*Hydrilla verticillate*, Alismatales: *Hydrocharitaceae*), filamentous algae (*Lyngbya wollei*, Cyanobacteria: Oscillatoriales), and duckweed (*Lemna minor*, Alismatales: *Araceae*) are widespread in the Everglades Agricultural Area (EAA) of South Florida. This situation is especially prevalent during the late spring to early fall when warm temperatures promote the growth of the aquatic weeds. Within the EAA, the total drainage water conveyance area that is not subjected to weed control can be covered on average by up to 50% of aquatic weeds. This coverage drops to 20% in the areas where aggressive aquatic weed control is applied [1]. Although floating aquatic weeds are capable of sequestering and storing high concentrations of phosphorus (P) from the drainage water and sediment, they release this nutrient back into the ecosystem once they senesce and die [2,3]. Large populations of floating aquatic weeds can reduce light penetration to the deep layer of the waterbody, thus resulting in a reduced primary production [4]. Moreover, excessive presence of aquatic weeds in farm canals can block water outlets during irrigation, clog canals, and impede water flow, resulting in low oxygen and poor water quality [5].

Allelopathy is a biological phenomenon during which an organism releases allelopathic chemicals into the environment [6]. Allelochemical compounds can influence the germination, growth, survival, and reproduction of neighboring organisms, and these effects can either be beneficial or harmful. Biological functions that are affected include cell division, membrane permeability, respiration, and photosynthesis [7]. In the past decades, the potential applications of allelopathy in agriculture have been hypothesized and investigated. Pesticidal effects of several plants were demonstrated. The aqueous extract of neem (*Azadirachta indica*, Sapindales: *Meliaceae*) killed a wide range of harmful insects [8]. Water hyacinth reduced the feeding rate of an insect pest, *Spodoptera litura* (Lepidoptera: *Noctuidae*), and was considered as a potential bioinsecticide for the management of this insect [9]. Among 13 different *Chara* populations screened against microalgal strains including cyanobacteria and eukaryotic microalgae, 10 inhibited growth of cyanobacteria [10]. After one day, water lettuce and duckweed had a significant negative effect on germination and root growth of a weed, common lambsquarters (*Chenopodium album*, Caryophyllales: *Amaranthaceae*) [11]. These findings revealed that some plants, including aquatic weeds, contain effective allelopathic compounds that can possibly be used as biopesticides.

The overall purpose of this study was to investigate the allelochemical properties of six aquatic weeds from Florida towards 100 bacterial strains, an insect pest, and three terrestrial weeds. The six aquatic weeds were chosen because they are the most prevalent and invasive species in the EAA watershed. More specifically, the research objectives were to determine the effects of extracts of these weeds on (i) the growth of bacteria collected from plants and soil samples in Florida; (ii) the survival, growth and feeding preference of larvae of the fall armyworm (FAW, *Spodoptera frugiperda*, Lepidoptera: Noctuidae), an insect pest of corn in Florida; and (iii) the germination and growth of nutsedge (*Cyperus esculentus*, Poales: *Cyperaceae*), amaranth (*Amaranthus tricolor*, Caryophyllales: *Amaranthaceae*), and common ragweed (*Ambrosia artemisiifolia*, Asterales: *Asteraceae*), three terrestrial weeds in Florida.

## Materials and methods

### Plant materials

Entire plants of muskgrass, water hyacinth, water lettuce, hydrilla, filamentous algae, and duckweed were collected from multiple farm canals within the EAA of South Florida (26°39’58” N; 80°37’45” W) during summer of 2018. These canals were either located on University of Florida’s property or on private properties for which authors had permission to access and collect samples.

#### Preparation of powdered aquatic weeds and aquatic weed extracts

All six aquatic weeds were washed utilizing a 1.0-L beaker and deionized (DI) water. The beaker was filled with aquatic weeds to the 0.5 L mark, then DI water was added until the resulting mixture reached the 1.0 L mark. The mixture was then stirred 10 times; after which the DI water was disposed. This process was completed twice, each time utilizing fresh DI water. The plants were then dried in a hot room at 50°C for 7 days until they were completely dry. The dried materials were finely ground to 1 mm particle-sized powder with a Thomas Model 4 Wiley laboratory mill and then stored in the dark in a plastic container at 25°C until further use.

Aquatic weed extracts were prepared using 5 g of powdered aquatic weed mixed with 50 ml DI water and 10 ml of 97% ethanol in a 200 ml wide-mouth conical flask. The mouth of the conical flask was sealed with aluminum foil, and the mixture was heated at 75°C for 4 hours in a Precision Thelco oven model 16. After heating, each flask was placed in a sonic bath for 1 hour and the aqueous residue was first filtered through cheese cloth followed by a Fisherbrand P5 5–10 μm filter paper. Ten to 30 ml of weed extract were obtained per batch. The nutrient composition of aquatic weed extracts and aqueous ethanol solvent was determined by the Soil, Water and Nutrient Management Laboratory of the University of Florida at Belle Glade (Table 1). Aquatic weed extracts used in bacterial growth assays described below were filtered through a 0.22 μm syringe filter (Millipore) before incorporation into the culture media.

**Table 1:** Nutrient composition content and pH (means ± SD) of six aquatic weed extracts and the aqueous ethanol control (as determined by the Soil, Water and Nutrient Management Laboratory of UF at Belle Glade).

		Elements (ppm)							pH
Extracts	Al	B	Ca	Fe	K	Mg	P	Si	
**Control**	0.05±0.03	0.09±0.05	2.09±0.40	0.04±0.01	1.63±0.11	0.57±0.06	0.14±0.04	0.10±0.04	7.23
**Muskgrass**	12.51±7.04	0.57±0.11	1517±1347	20.08±1.99	485.9±389.7	252.0±55.49	27.88±2.20	65.0±23.01	6.81
**Water hyacinth**	3.42±0.50	1.26±0.25	1409±966.1	6.39±1.41	636.8±528.2	236.6±9.00	43.79±28.14	44.83±14.5	5.97
**Water lettuce**	18.33±11.51	10.36±14.16	2070±1192	15.97±8.58	262.1±313.3	171.8±24.59	43.04±0.13	76.16±2.66	6.41
**Hydrilla**	15.55±3.05	0.59±0.16	1572±1426	20.82±3.54	502.1±424.7	256.1±54.64	28.30±3.14	64.99±22.02	6.85
**Filamentous algae**	3.37±0.46	1.21±0.30	1464±1044	6.51±1.66	628.7±515.7	238.3±8.46	44.21±28.38	48.43±14.93	7.18
**Duckweed**	19.89±10.77	10.97±15.16	2060±1215	16.19±8.93	263.2±315.3	174.7±26.56	43.35±0.66	79.81±4.05	7.12

### Media used for isolation and culturing of bacteria

The basic medium (BM) used to isolate and grow bacteria contained yeast extract (5 g), bactopeptone (5 g), sucrose (5 g), agar (15 g), and 1 L DI water. Medium pH was adjusted to 6.8–7.0 with the Fisher Scientific buffer solution pH 10.00 or pH 4.00 before autoclaving the medium at 120°C for 20 minutes. After autoclaving, when the agar medium had cooled down to around 50–60°C, 0.1 g cycloheximide mixed in 5 ml of 97% ethanol solution and 1 μl Tilt^®^ 250 EC fungicide (propiconazole, Syngenta Crop Protection, Greensboro, NC) were added and mixed evenly. Approximately 20 ml of the BM supplemented with the anti-fungal compounds were poured into sterile Petri dishes of 10 cm diameter, and maintained at room temperature under a laminar hood until solidification of the medium. Blank plates contained BM supplemented with the aqueous ethanol solvent used to prepare aquatic weed extracts: 10 ml and 1 ml of the 16.17% ethanol solution was added to 1 L of BM after autoclaving to obtain a final solvent dilution of 1:100 and 1:1,000, respectively. Extract plates contained BM supplemented with one of the six microfiltered aquatic weed extracts (muskgrass, water hyacinth, water lettuce, hydrilla, filamentous algae, and duckweed): 10 ml and 1 ml of a plant extract were added to 1 liter of BM after autoclaving to obtain a final weed extract dilution of 1:100 and 1:1,000, respectively.

#### Isolation and storage of bacteria

During fall 2018, 100 bacterial strains were isolated from 25 plants (sweet corn, sorghum, papaya, etc.) and soil samples collected from the Everglades Research and Education Center (EREC) at Belle Glade and other locations in Florida (Milton, Gainesville, Ft. Pierce, and Homestead) (S1 Dataset). Plants for isolation of bacteria were collected on University of Florida property or on public domain where no sampling permits were required. Under a laminar floor hood, an approximately 2-cm^2^ area of each leaf sample was cut in a Petri dish into small pieces in 1 ml of sterile DI water. Approximately 0.5 g of soil sample was added into a 14-ml polystyrene tube and mixed with 8 ml of sterile DI water. One hour after homogenization of the leaf or soil material in water, a drop of liquid was transferred with a smear loop to a plate containing BM. The liquid sample containing bacteria was spread evenly on the medium with the smear loop before sealing plates with parafilm (M PM996 All-Purpose Laboratory Parafilm Film). Plates were incubated at 28°C for 2–3 days. For each plant or soil sample, single colonies with different colors or different growth rates were sub-cultured on fresh BM plates. Once bacteria were sub-cultured successfully, each BM plate was inoculated with 16 bacterial isolates.

For storage of the bacterial collection, a bacterial suspension (>10^10^ colony forming units/ml) was prepared for each isolate in a 2 ml tube containing 0.5 ml of 50% glycerol and 1.5 ml sterile DI water. Each bacterial suspension was stored in a freezer at -80°C until further use.

#### Effect of weed extracts on bacterial growth

Each bacterial isolate was streaked with an inoculation loop on three media: BM, BM + solvent (1:100 and 1:1,000 dilution) and BM + weed extract (1:100 and 1:1,000 dilution). BM was considered the positive control growth medium, BM + solvent was the blank medium without weed extract, and BM + weed extract was the testing medium. Each plate was inoculated with 16 bacterial isolates. All the plates were incubated at 28°C for three days and growth of bacterial cultures was observed after this incubation time.

When bacteria did not or only partially grew on the testing medium (corresponding to possible growth inhibition), a dilution plating method was performed. This method allowed us to determine the concentration (number of colonies) of an unknown sample by counting the number of colonies cultured from serial dilutions of the sample, and then back track the measured counts to the unknown concentration [12].

**Fig 1:**
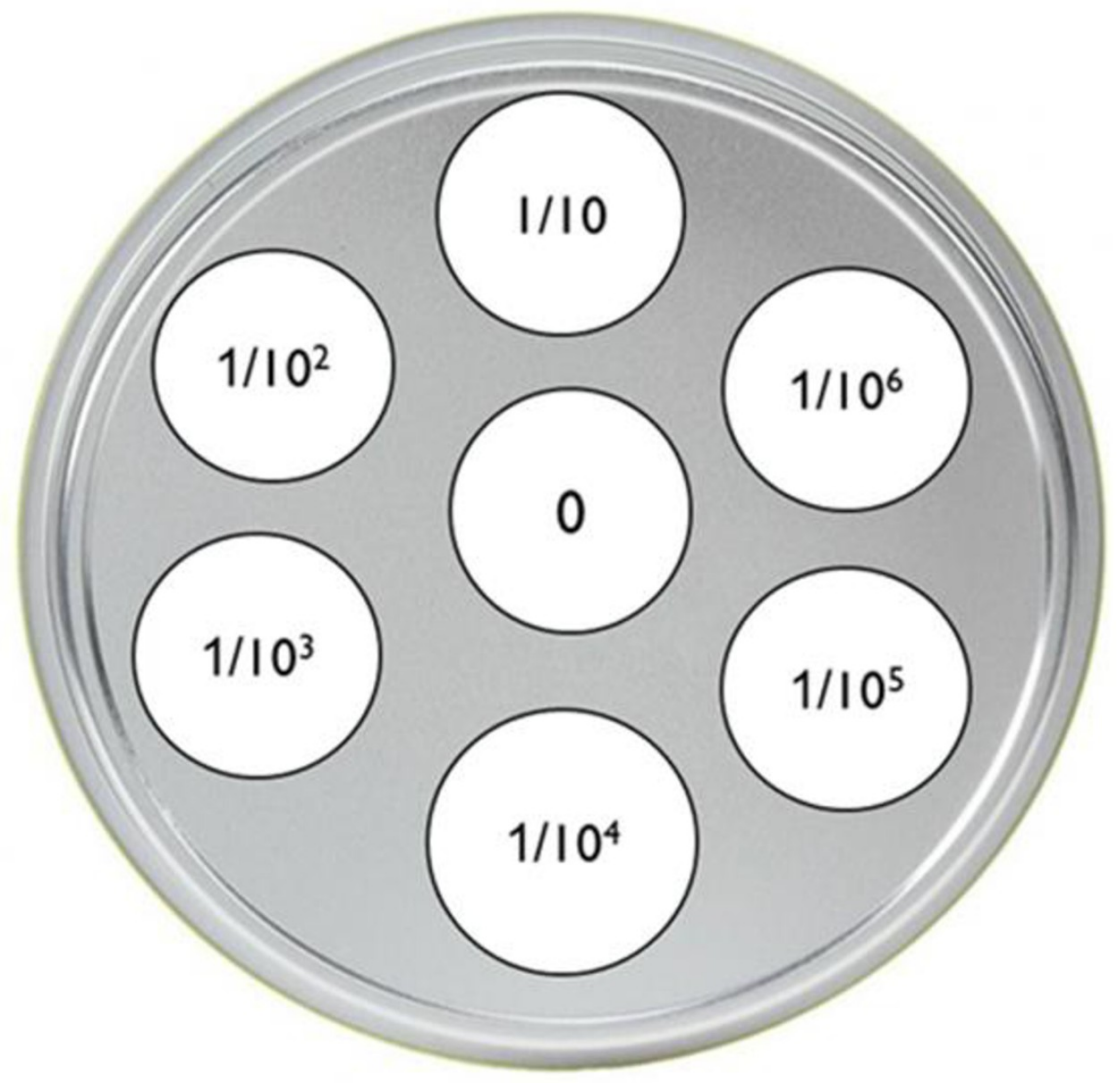
Schematic aspect of a serial dilution plate to determine the concentration of a bacterial suspension. 0 = 50 μl drop of undiluted bacterial suspension; 1/10-1/10^6^ = 1/10-1/10^6^ diluted drops of a bacterial suspension.

The first step was to take a single bacterial colony of stock and to place it into 0.9 ml DI water, and this was the original stock. The technique used to make a single dilution was repeated sequentially using more and more diluted suspensions, at each step: 0.1 ml of the previous suspension was added to 0.9 ml of DI water, each step resulting in a further 10-fold change in the concentration from the previous concentration. The first tube after the original stock was a 1:10 dilution, the second a 1:100, the third a 1:1,000, etc., to the sixth, a 1:10^6^ dilution. The second step was to determine the number of viable cells in each of the dilutions. A pipette was used to transfer 50 μl of each dilution, starting from 1:10^6^ (the most diluted) to 1:10 and finally from the original stock onto an agar plate of each of the three tested media (Fig 1).

The plates would have different numbers of colonies depending on the dilution of the sample. When too many colonies grow, it can be very difficult to count them. Thus, we counted the number of colonies only for dilutions for which less than 50 colonies grew. The bacterial growth was considered to be partially inhibited when the bacterial colony number on BM + weed extract at 1:10^n^ dilution was more than 50 percent less than the bacterial colony number on BM at 1:10^n-1^ dilution.

### Insect materials

7-day old FAW larvae obtained from a colony maintained in the laboratory at the EREC were used in biossays. The colony was initiated in October 2018 using approximately 150 larvae collected in the field on sweet corn at the EREC. The colony has been maintained in a climate-controlled room at 25.5±1°C, 40–60% RH (relative humidity), and L14:D10 h (light: dark hour) photoperiod. Larvae were individually kept in 37-ml translucent plastic cups (Dart Container Corp., Mason, MI) filled with Stonefly Heliothis Diet (Ward’s Science, Rochester, NY). Upon larval development completion, 15–30 pupae were placed in moist vermiculite at the bottom of a 3.7-L carton container lined with cheesecloth and paper towels. Adult eclosion and oviposition was monitored 3 times a week, and cheesecloth and paper towels with FAW eggs were placed in transparent plastic bags. Newly emerged neonate larvae were transferred using a paintbrush from plastic bags to cup with diet until pupation or use in bioassays.

#### Experimental treatments

The organic insecticides used as commercial standards were pyrethrins (PyGanic Crop Protection 5.0 ECII, Valent U.S.A., Walnut Creek, CA) for larval dip bioassays and *Bacillus thuringiensis subsp*. *kurstaki* (Bt, Dipel DF, Valent BioSciences, Libertyville, IL) for larval diet incorporation bioassays. PyGanic 5.0 ECII is registered for foliar applications in the United States at 329–1,242 ml/ha and a concentration of 4.2 ml/L was used in this study. Dipel DF is registered for application in the United States at 560–2,242 g/ha and a dosage of 7.5 g/L was used in this study. In addition to commercial standards, bioassays were conducted with six aquatic weed extracts and one control solution at 1:10 dilution.

#### Effect of weed extracts on FAW survival and growth

Laboratory experiments were conducted to determine the effect weed extracts on FAW larval survival and growth. Contact activity of the extracts was determined in larval dip bioassays, whereas ingestion activity was determined in larval diet incorporation bioassays. Each type of bioassay was conducted four times using two different FAW generations and two different FAW cohorts two days apart per generation in order to reduce the impact of a specific cohort on the results. A total of 130 FAW larvae (replications) were used for each treatment in each type of bioassay (50 larvae per treatment for each of the two cohorts of the first generation and 15 larvae per treatment for each of the two cohorts of the second generation). FAW larvae were starved overnight to void their gut and then weighed immediately on a TS400D Precision Standard Balance (Ohaus Corp., Parsippany, NJ) before each bioassay.

For larval dip bioassays, each larva was individually dipped for 1 second in the treatment solution. The larva was subsequently placed on filter paper for 10 seconds, and then in a cup with diet. Dipped larvae were reared in the insectary for 4 days. For larval diet incorporation bioassays, each larva was placed in a cup with treated artificial diet prepared with a treatment solution. Transferred larvae were reared in the insectary for 4 days. Larvae were considered alive if they could right themselves after being flipped on their back and prodded for a maximum of 10 seconds with the blunt tip of a metallic pin. The live larvae were subsequently starved overnight and weighed to determine their relative growth rate (RGR). The RGR of a larva is its daily growth rate relative to its average size over the duration of an assay. The RGR was calculated for each live larva using the following formula:

$$RGR=\frac{Final\;weight-Initial\;weight}{(Initial\;weight+Final\;weight/2)*Feeding\;duration}$$

with weight expressed in grams and feeding duration expressed in days [13].

#### Effect of weed extracts on FAW feeding preference

**Fig 2:**
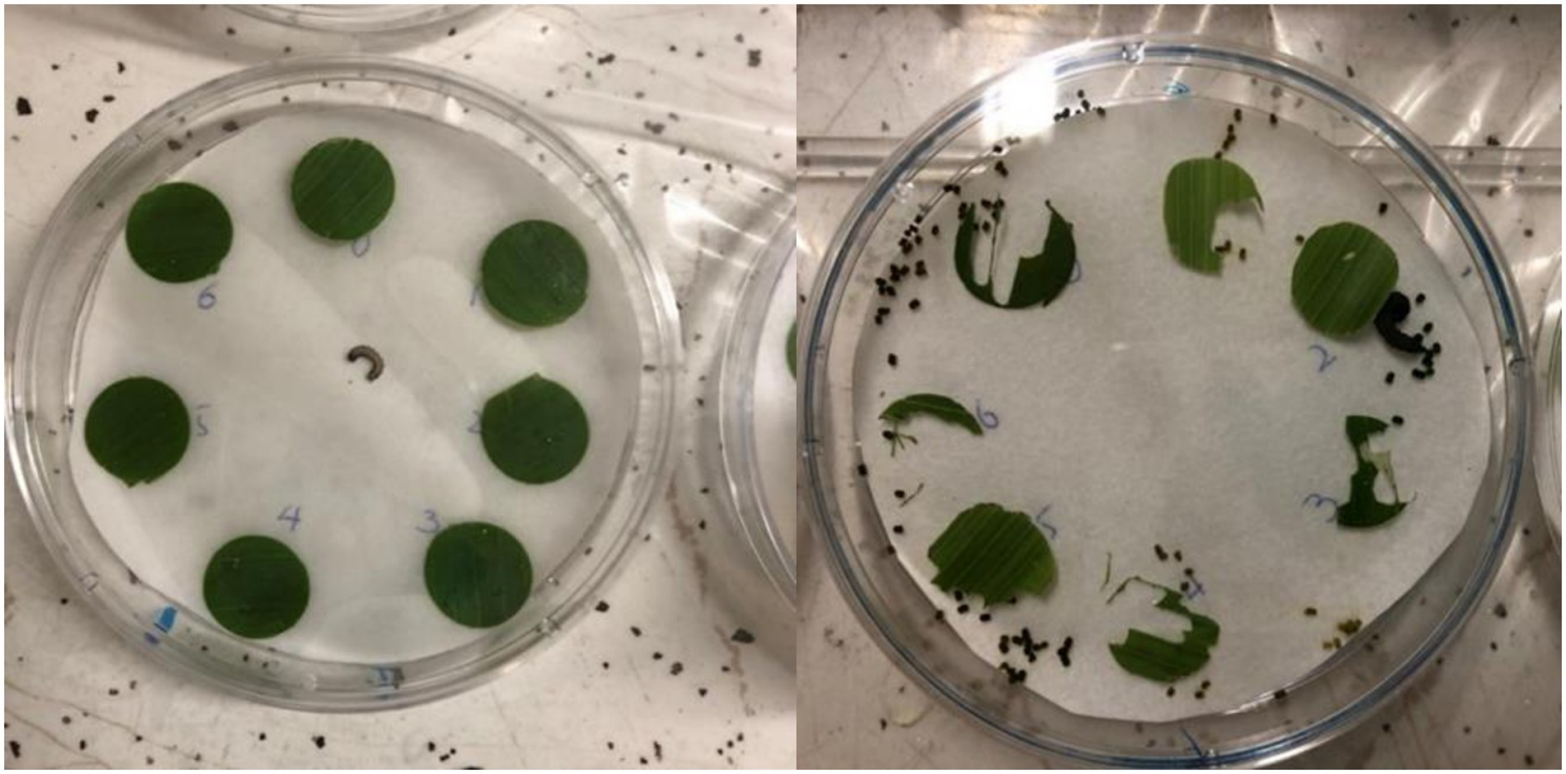
Leaf disc before and after 24 hours of fall armyworm release.

A laboratory experiment was conducted to determine the effect of aquatic weed extracts on FAW larval feeding preference. This experiment consisted of a choice bioassay conducted twice using two different FAW and sweet corn plant cohorts. For this experiment, we used plastic Petri dishes (15 mm x 100 mm diameter, Cole-Parmer Instrument Company, LLC, Vernon Hills, IL). A total of 40 Petri dishes (replications) were prepared (20 per bioassay). For each bioassay, 2-week old sweet corn plants (‘Cabo’ hybrid, Syngenta, Greensboro, NC) grown in a greenhouse were randomly selected and leaves were cut into leaf discs with a disc cutter (1.6 cm in diameter). Each leaf disc was dipped for 1 second into the aquatic weed extract or the control solution. The Petri dishes were lined with one layer of wet filter paper to prevent leaf disc and FAW larva desiccation. The treated discs were placed on filter paper to remove the excess liquid, and then in the Petri dishes. Each Petri dish included all experimental treatments along a circular layout. One FAW larva was placed at the center of each Petri dish. All the Petri dishes were kept in the climate-controlled room and subsequently observed 24 and 48 hours after the FAW larva was released (Fig 2). Leaf area was measured for each leaf disc by using the mobile application LeafByte as described in [14].

### Terrestrial weed materials

During fall 2019, the tubers of nutsedge (*Cyperus esculentus*) and the seeds of amaranth (*Amaranthus tricolor*) and common ragweed (*Ambrosia artemisiifolia*) were collected in the field at the Southwest Florida Research and Education Center (SWFREC) in Immokalee, FL.

#### Effect of weed extracts on terrestrial weed germination and growth in greenhouse

In the greenhouse, for each species of terrestrial weed, 10 seeds or tubers were planted 1–2 cm deep in 1-L plastic pots. The soil used for planting was Black Gold all-purpose potting mix with a pH of 5.5–6.5 and N-P-K of 0.13–0.04–0.13 (www.sungro.com). Each pot was filled with approximately 340 g of potting mix. Four rates (0, 0.1, 0.5, and 1.0 g) of ground powder of aquatic weeds were added on the surface of the pot (103 cm^2^) and mixed evenly with top soil. Pots with no aquatic weed extract were used as control. Four replications were prepared for each treatment and each application rate. All the pots were kept in the greenhouse at 27°C and 50% RH, and sprinkle-watered every day to the field capacity of each pot. The sprinkler was set to mist level to avoid the leaching of aquatic weed powder by the heavy drops of water. The germination rate of plants in each pot was recorded for five weeks after planting (days 8, 12, 18, 22, and 39). Visible coleoptiles on the soil surface were used as an indicator of plant emergence. Data were determined as the total number of germinated seeds/tubers divided by the total number of seeds/tubers in each pot. After six weeks, the fresh biomass weight of each pot was assessed by cutting plants at the soil level and weighing them using a laboratory scale (Ohaus; model: PX124).

#### Effect of weed extracts on terrestrial weed germination in laboratory

The effects of different dilutions of the aquatic weed extract solutions (dilution at 1:100 and 1:10) were studied using a laboratory bioassay. Five tubers of nutsedge, 10 seeds of amaranth and common ragweed were germinated in a 100 mm diameter polystyrene Petri dish lined with two layers of 90 mm size Whatman 1 filter paper. The filter paper was moistened with 5 ml of each extract solution. The same amount of aqueous ethanol solution was used for the aqueous ethanol control, and the RO water solution was used as water control. Four replications were prepared for each treatment and each dilution. All Petri dishes were sealed with PM996 All-Purpose Laboratory Parafilm to reduce the loss of water and then maintained in a growth chamber with light from 7 am (25°C, 50% RH) to 7 pm. The temperature of the growth chamber at night was 22°C with 50% RH. All Petri dishes were randomly distributed in the growth chamber. Visible coleoptiles on the soil surface were used as an indicator of plant emergence. Germination values were calculated by dividing the total number of germinated seeds or tubers by the total number of seeds or tubers in the Petri dish. Germination values were determined after one week of growth.

### Statistical analysis

For the antimicrobial streaking assay, the evaluation of bacterial growth was only qualitative (yes or no or weak) and not quantitative. These qualitative data can therefore not be statistically analyzed. The second assay performed with three isolates was based on quantitative data (colony counts) and has been statistically analyzed using a one-way ANOVA test.

Statistical analyses were conducted using SAS PROC GLIMMIX [15]. For the larval dip and diet incorporation bioassays, linear mixed models were applied to compare survival rate and RGR of FAW as affected by treatment. Treatment was a fixed effect, whereas generation and cohort(generation) were random effects (S1 Dataset). For the leaf disc assays, linear mixed models were used to compare leaf area consumed by FAW larvae as affected by treatment and observation time. Treatment, observation time, and their two-way interaction were fixed effects. Assay, dish(assay), and treatment x dish(assay) were random effects (S1 Dataset). Thus, a variance component covariance structure was used to model the effects of repeated measures of the leaf disc assays. The Kenward-Roger adjustment for denominator degrees of freedom was used to correct for inexact F distributions [15]. Least square means ± SEs from the least square means statement output are reported unless stated otherwise. The Tukey-Kramer adjustment (Alpha = 0.05) was used for pairwise separation of least-square means when a fixed effect was detected (P<0.05).

Statistical analyses were conducted using SAS PROC GLM to compare germination rates and biomass affected by each treatment rate of the powdered aquatic weed experiment [16]. For the aquatic weed extract experiment, the PROC GLM procedure was used to compare germination rates affected by each treatment and dilution. Pairwise mean separation was performed when using the Tukey-Kramer adjustment (P ≤ 0.05) [16].

## Results and discussion

### Antimicrobial activity of aquatic weeds

A total of 100 different bacterial strains were isolated from 18 plant and 7 soil samples. After culturing these bacteria on culture medium with six different weed extracts, the growth of 97 of them was not affected by any of the six aquatic weed extracts. Only three of the 100 strains showed a partial growth on the medium with extracts at 1:100 dilution. These three strains included isolate 11 and isolate 72 on BM + muskgrass extract, isolate 58 on BM + water hyacinth extract, and BM + filamentous algae extract. After further testing of these isolates by the dilution plating method (Table 2), the bacterial colony counts for isolate 11 on BM + muskgrass were not significantly different (P = 0.386) from the colony counts on the control plates. Similarly, colony counts for isolate 72 on BM + muskgrass and for isolate 58 on BM + water hyacinth were not different from those on their respective control plates (P = 0.540 and P = 0.056). Only bacterial counts for isolate 58 on BM + filamentous algae were significantly lower (P = 0.013) compared to the control plates (54% growth reduction). This suggested that the partial growth observed in the first assay for these isolates (with the exception of isolate 58 on filamentous algae extract) was due to insufficient inoculum during streaking and not an inhibitory effect of the culture medium. Consequently, there was overall no visible difference in bacterial growth of the 100 bacterial strains on plates containing weed extracts and on control plates. These results suggested that the six aquatic weed extracts did not contain any antibacterial compounds or only at very low concentrations, or that biologically active molecules were inactivated during the extraction process.

**Table 2:** Comparison of bacterial colony numbers (colony forming units/ml or cfu/ml) of three bacterial isolates on dilution plates of basal medium (BM) and BM supplemented with aquatic weed extracts (1:100 dilution).

Isolate No.	Bacterial colony number (cfu/ml) on				P value
	Control (BM)	BM + Muskgrass	BM + Water hyacinth	BM + Filamentous algae	
11	16±3 at 10^−4^ (a)	13±5 at 10^−4^ (a)	ND	ND	0.386
58	37±7 at 10^−4^ (a)	ND	21±5 at 10^−4^ (a,b)	17±1 at 10^−4^ (b)	0.011
72	12±4 at 10^−2^ (a)	15±8 at 10^−2^ (a)	ND	ND	0.540

ND = Not determined

Values are the means of three plate counts. For each isolate and on each line, values followed by the same letter in parentheses are not significantly different at P = 0.05 (One-way ANOVA).

In a similar study, water lettuce was shown to have an antimicrobial activity based on inhibition zones of *Escherichia coli*, *Bacillus subtilis*, and *Klebsiella pneumonia* [17]. However, only fresh samples of water lettuce had a growth inhibitory effect, whereas an aqueous extract did not show this activity [17]. Antibacterial activity of medicinal plants after extraction with different solvents suggested that extractions made with acetone and methanol were more efficient than water to recover molecules with antibacterial activity [18]. Methanol might be more effective at recovery of higher concentrations of antibacterial compounds [18]. Abraham et al. tested antibacterial activity of different extracts of water lettuce using clinical pathogens [19]. Compared to chloroform and hexane, methanol extracts resulted in higher yields of chemicals such as alkaloids, phytosterols, phenols, flavonoids and tannins that possess bioactive properties. Tripathi et al. came to the same conclusion after testing a methanolic extract of water lettuce that showed stronger antimicrobial activity than a hexane extract [20].

The studies mentioned above confirmed the hypothesis that aquatic weeds can contain allelochemicals with antimicrobial properties. These studies also showed that the methods of extraction are critical to obtain a positive result. The negative results obtained in our study can be associated with the extraction method or the bacteria used for testing. Therefore, in the future, instead of using water and ethanol extracts, different organic solvents such as methanol and acetone could be investigated to identify antibacterial compounds in aquatic weeds of the EAA. Furthermore, we tested diverse bacteria isolated from an agricultural environment. Another option could be to test bacteria from a clinical environment.

### Insecticidal activity of aquatic weeds

For the larval dip bioassay, differences in survival rate were not detected among treatments, including the control (P = 0.994) and commercial treatment (P = 0.979; Table 3). However, differences in RGR were detected among treatments (P < 0.001). The highest RGR, 0.415 g g^-1^ day^-1^, was observed with filamentous algae and this RGR value was 1.05-fold higher than the RGR values for the commercial treatment and muskgrass (P < 0.001). However, none of the treatments were different than the control (muskgrass: P = 0.951; water hyacinth: P = 0.828; water lettuce: P = 1.0; hydrilla: P = 0.987; filamentous algae: P = 0.164; duckweed: P = 0.829). Therefore, the dermal contact of aquatic weed extracts did not have an apparent effect on FAW survival or growth. It was unexpected for the commercial pyrethrins to not affect FAW, but according to Abrahams et al., FAW has become resistant to insecticides in modes of action groups 1A (carbamates),1B (organophosphates), and 3A (pyrethroids and pyrethrins) in several parts of the Americas, thus higher doses or alternative chemicals or methods have to be applied [21]. This may explain why the commercial treatment used in our study was not effective against FAW.

**Table 3:** Survival rate and RGR of the fall army worm in dip bioassays with different treatments.

Treatment	Survival rate (%)	RGR (g g^-1^ day^-1^)
**Muskgrass**	98± 1.38 a	0.396 ± 0.02 b
**Hyacinth**	98 ± 1.38 a	0.409 ± 0.02 ab
**Water lettuce**	99 ± 1.38 a	0.402 ± 0.02 ab
**Hydrilla**	99 ± 1.38 a	0.406 ± 0.02 ab
**Filamentous algae**	96 ± 1.38 a	0.415 ± 0.02 a
**Duckweed**	99 ± 1.38 a	0.409 ± 0.02 ab
**Control**	99 ± 1.38 a	0.402 ± 0.02 ab
**Commercial**	98 ± 1.38 a	0.395 ± 0.02 b
**F**	0.70	3.58
**df**	7,1029	7,1009
**P>F**	0.672	< 0.001

Means in a column followed by the same letter are not significantly different (Tukey-Kramer adjustment, alpha = 0.05).

For the larval diet incorporation bioassay, average survival ranged from 5.5 to 93%. Differences in survival were detected among treatments (P < 0.001; Table 4). Survival in the commercial treatment was 93 to 94% lower than in any other treatment (P < 0.001), including the control. Survival in water lettuce was 14% lower (P < 0.001) than in the control, but also 12% lower than in hydrilla, and 15% lower than water hyacinth. All other survival rates of aquatic weed extracts were not significantly different from the control (muskgrass: P = 0.928; water hyacinth: P = 1.0; hydrilla: P = 1.0; filamentous algae: P = 0.132; duckweed: P = 0.829). The RGR of FAW larvae in the diet incorporation bioassay differed among treatments (P < 0.001; Table 4). The commercial treatment had a negative RGR (-0.0215 g g^-1^ day^-1^), which was lower than any other RGRs determined in the bioassay. This observation indicated that in general, the larvae that survived this treatment lost weight. However, hydrilla caused 11.3% reduction in RGR compared to the control (P < 0.001). The RGR of larvae feeding on the diet with hydrilla was also significantly lower than for water lettuce (1.1-fold; P < 0.001) and water hyacinth (1.1-fold; P < 0.001). Duckweed reduced RGR of larvae by 9.4% (P < 0.001) relative to the control. The RGR associated with duckweed was also 8.9% lower than water lettuce (P < 0.002), and 11.3% lower than water hyacinth (P < 0.001). The RGR for other aquatic weed extracts were not significantly different from the RGR of the control (muskgrass: P = 0.791; water hycinath: P = 0.994; water lettuce: 1.0; filamentous algae: P = 0.554). *B*. *thuringiensis* showed a strong insecticidal effect on FAW larvae with high mortality and low RGR. The average value of RGR was negative, which indicated that the larvae that survived generally lost weight and lived in a very poor health after consuming *B*. *thuringiensis*. This result is consistent with the previously reported effectiveness of insecticidal properties of toxins produced by this bacterial species [22]. Water lettuce showed a negative impact on FAW larvae survival, and hydrilla and duckweed demonstrated reduction in FAW larvae growth. A chemical analysis by Tripathi et al. revealed that the biologically active chemical constituents of water lettuce are alkaloids, glycosides, flavonoids, tannins and steroids [23]. Hydrilla contains phenolic and hydroxy acid. Loliolide was also detected in ethyl acetate and ethanol extracts [24,25]. An earlier study identified tannins as inhibitor of the growth of various species of pest insects including Lepidoptera larvae. Those tannins can impede the activity of proteases and thus result in negative effects on larvae health and growth [26]. Loliolide was reported to have diverse biological properties including repellency against insects [27].

**Table 4:** Survival rate and RGR of the fall armyworm in different treatments (a negative value indicates that the FAW lost weight during the bioassay).

Treatment	Survival rate (%)	RGR (g g^-1^ day^-1^)
**Muskgrass**	89 ± 8.73 ab	0.366 ± 0.03 ab
**Water hyacinth**	93 ± 8.73 a	0.389 ± 0.03 a
**Water lettuce**	79 ± 8.73 b	0.379 ± 0.03 a
**Hydrilla**	91 ± 8.73 a	0.339 ± 0.03 b
**Filamentous algae**	84 ± 8.73 ab	0.362 ± 0.03 ab
**Duckweed**	88 ± 8.73 ab	0.345 ± 0.03 b
**Control**	92 ± 8.73 a	0.381 ± 0.03 a
**Commercial**	5.5 ± 8.73 c	-0.022 ± 0.04 c
**F**	174.31	48.71
**df**	7,1028	7,843.7
**P>F**	< 0.001	< 0.001

Means in a column followed by the same letter are not significantly different (Tukey-Kramer adjustment, alpha = 0.05).

In the leaf disc bioassays, the percentage of leaf area over the two observation times was between 67% and 77% among treatments, but there was no difference among treatments including the control (F = 0.87; df = 6,234; P = 0.517; Table 5). There was a significant difference between the two observation times (F = 162.51; df = 1,273; P < 0.001). An interaction between treatment and observation time was not detected (F = 1.06; df = 1,273; P = 0.388). Thus, the feeding activity of FAW larvae was not affected by any of the treatments in our study. Compounds that possess antifeedant activity can be found in all chemical classes in plants [28]. Terpenes and alkaloids are the two classes that are especially effective in inhibiting feeding of a variety of insects. In a similar study, the leaves treated with methanol and n-hexane extracts of water hyacinth suggested high antifeedant efficacy on *Spodoptera litura* [9]. More than 20 types of chemical compounds (including alkaloids, phenols and flavonoids) with potential insecticidal activity were identified by gas chromatography-mass spectrometry (GC-MS) analysis of methanol extract of water hyacinth [29]. This suggested that some other organic solvents such as methanol and n-hexane might be efficiently used to obtain higher yields of effective compounds from aquatic plants against insect larvae.

**Table 5:** Leaf area consumed by FAW larvae 24 and 48 hours after application of different treatments in leaf disc bioassays.

	Leaf area consumed (%)	
Treatment	After 24 hours	After 48 hours
**Muskgrass**	68 ± 9.96	85.70 ± 9.96
**Water hyacinth**	59 ± 9.96	89.13 ± 9.96
**Water lettuce**	54 ± 9.96	85.28 ± 9.96
**Hydrilla**	52 ± 9.96	80.58 ± 9.96
**Filamentous algae**	56 ± 9.96	77.53 ± 9.96
**Duckweed**	60 ± 9.96	80.13 ± 9.96
**Control**	61 ± 9.96	88.45 ± 9.96

There were no significant differences among treatments (Tukey-Kramer adjustment, alpha = 0.05).

Overall, water lettuce, hydrilla and duckweed showed significant effects on FAW larvae survival and growth by ingestion. However, the observed differences were small, and none of the aquatic weeds possessed contact and antifeedant effect towards FAW larvae. In the future, different solvents can be used to isolate higher contents of the allelopathic compounds in the aquatic weed that are likely to have better insecticidal activity.

### Herbicidal activity of aquatic weeds

**Fig 3:**
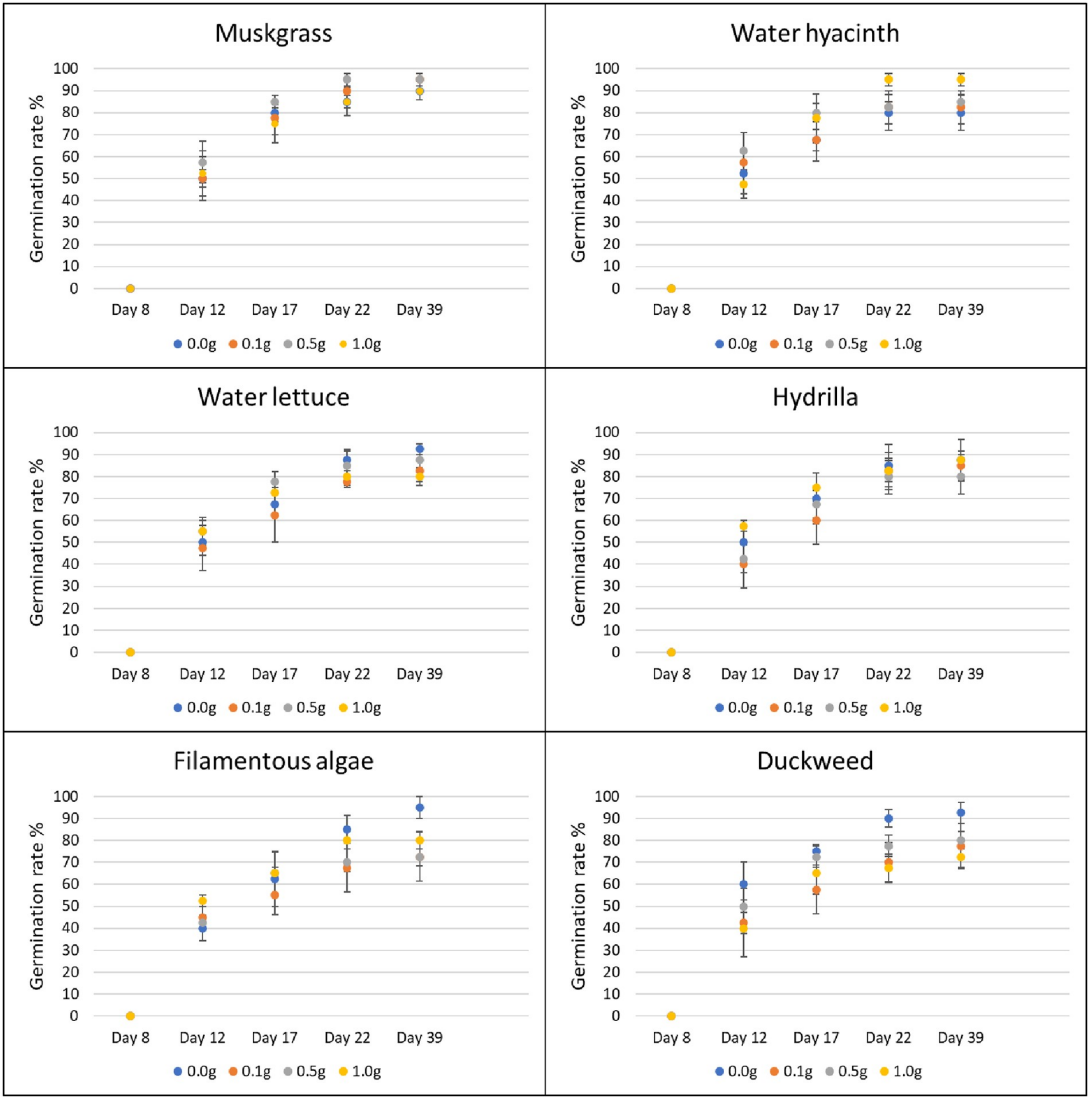
Effect of aquatic weed powder on germination rate of common ragweed. Vertical bars represent standard errors.

**Fig 4:**
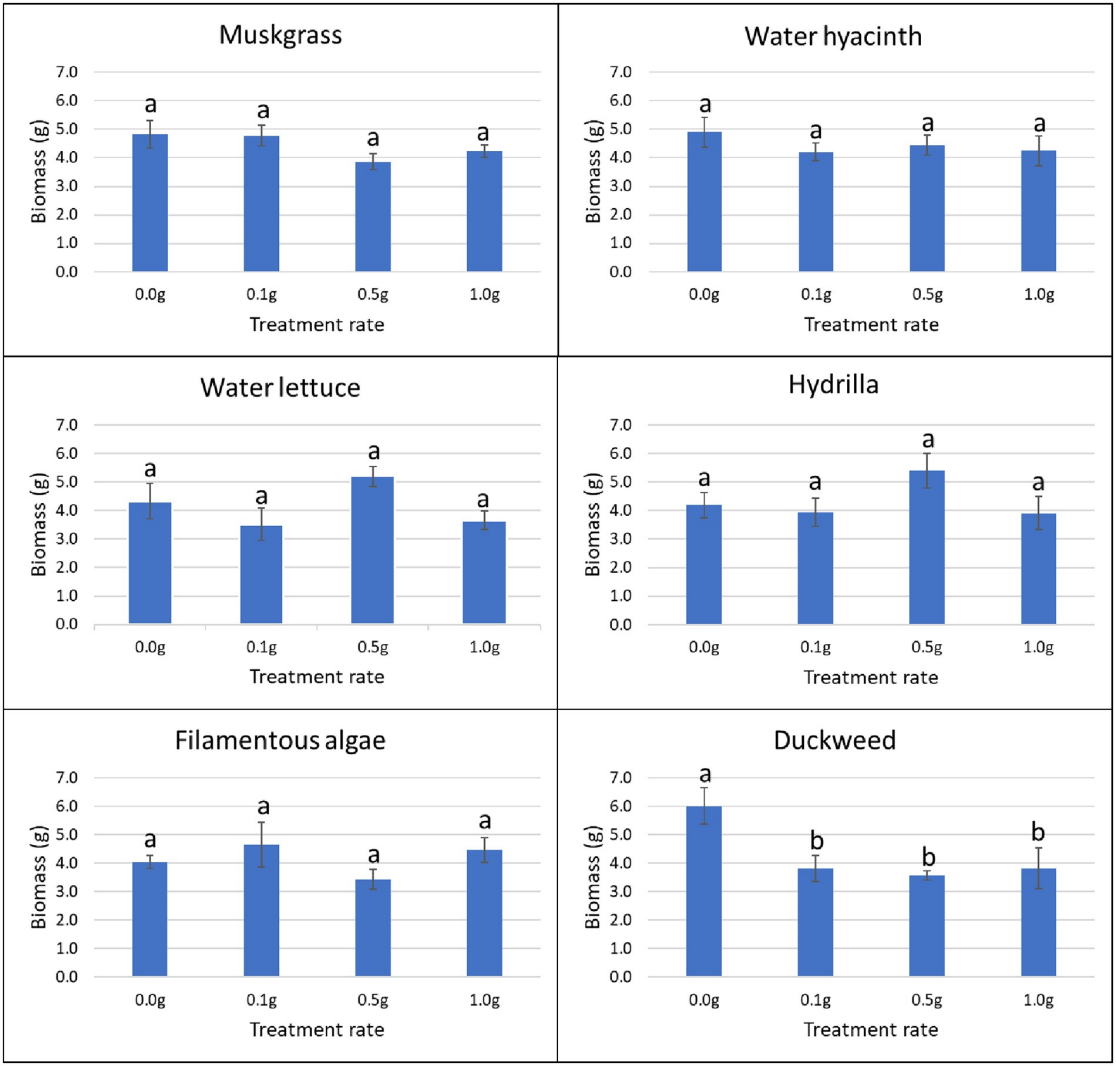
Effect of aquatic weed powder on biomass of nutsedge. Vertical bars represent standard errors of the mean. Bars designated by different lowercase letters are significantly different according to Tukey-Kramer adjustment (P ≤ 0.05).

In the greenhouse, application of duckweed powder resulted in a growth reduction of nutsedge. At the rate of 0.1 g, 0.5 g and 1.0 g, powder of this aquatic weed reduced the biomass of nutsedge by 36.6%, 40.5% and 36.3%, respectively (P = 0.042). The other aquatic weed powders did not have any effect on nutsedge germination or growth as compared to the control (Figs 3 and 4).

**Fig 5:**
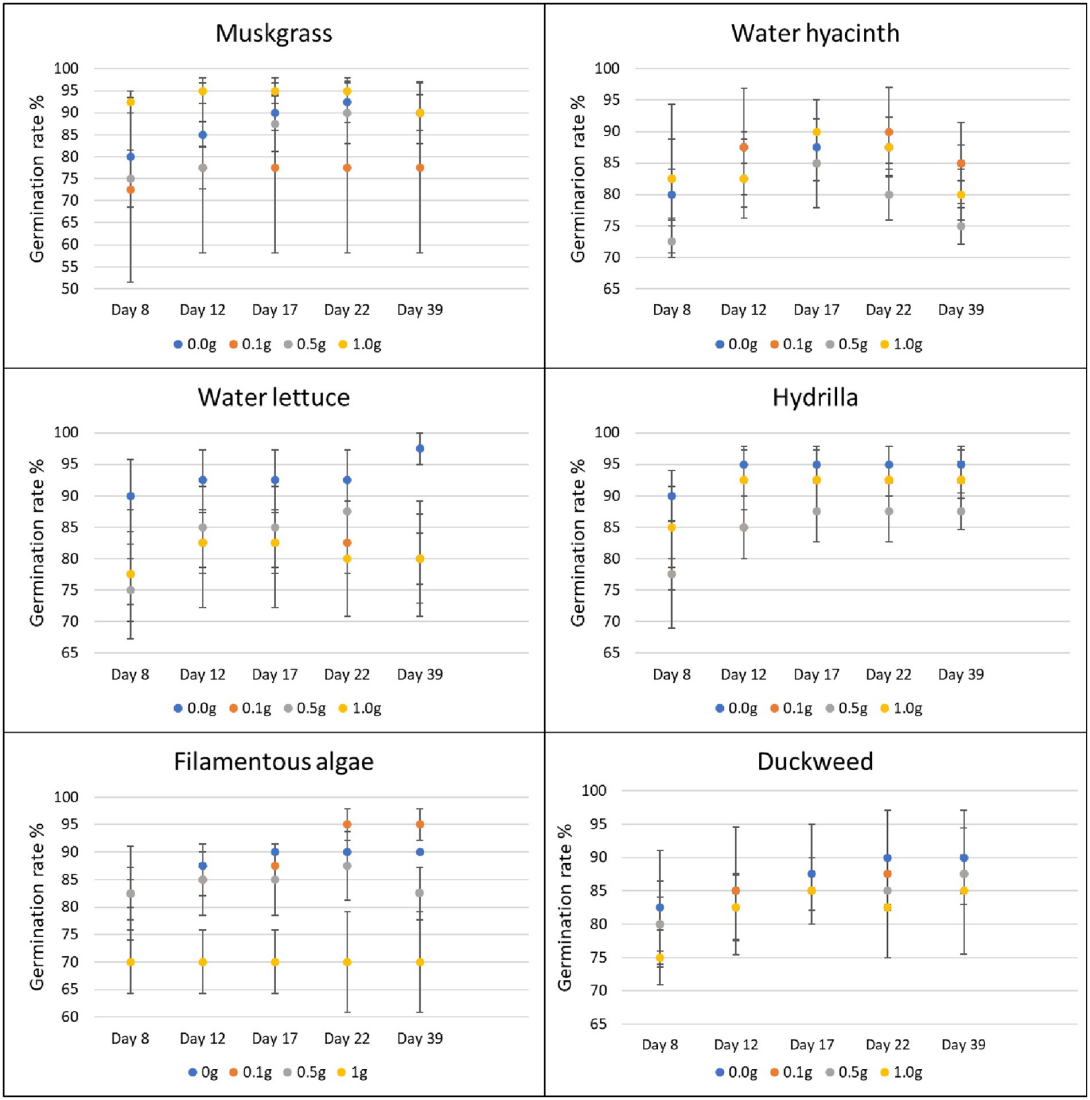
Effect of aquatic weed powder on germination rate of amaranth. Vertical bars represent standard errors.

**Fig 6:**
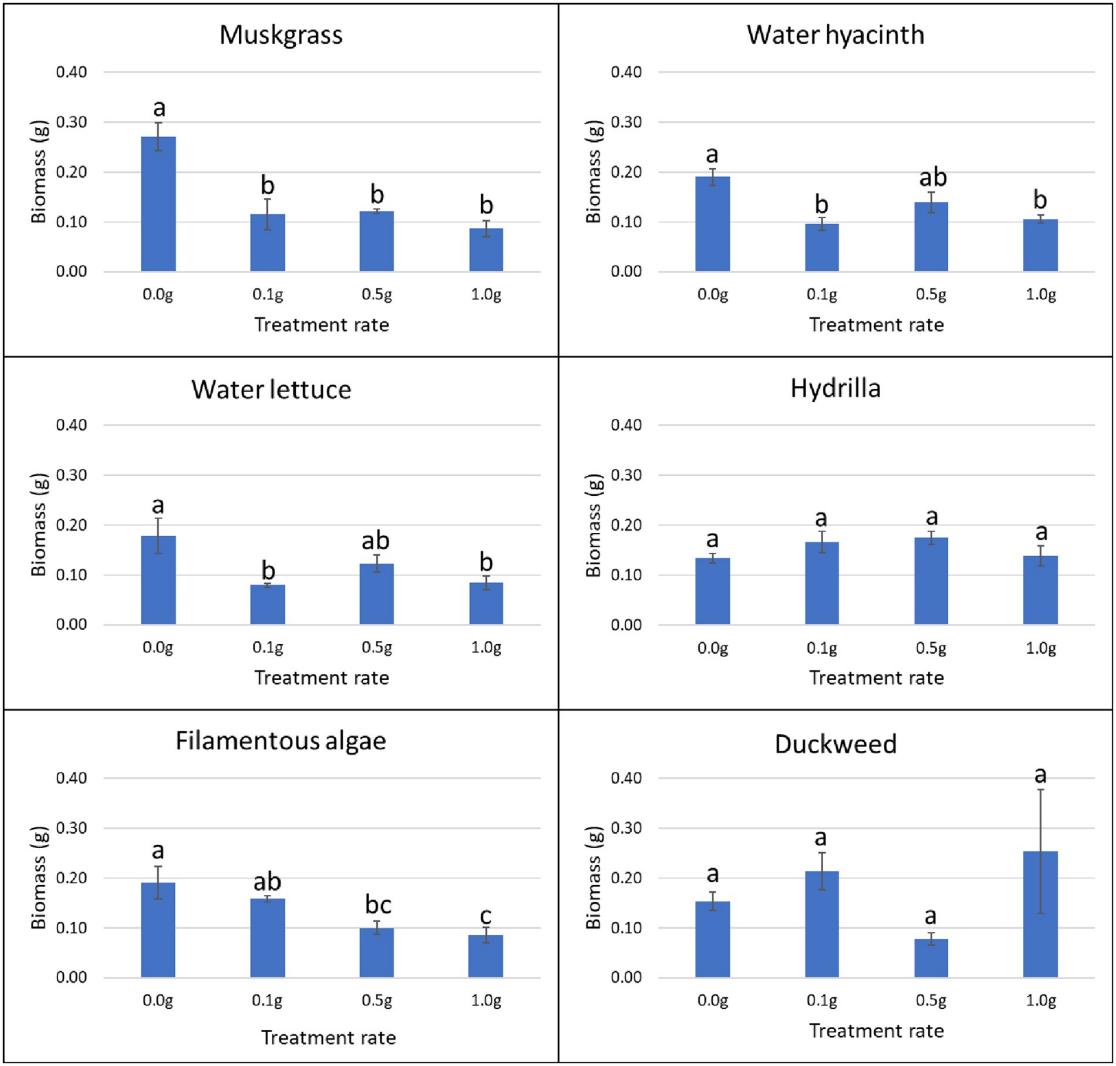
Effect of aquatic weed powder on biomass of amaranth. Vertical bars represent standard errors of the mean. Bars designated by different lowercase letters are significantly different according to Tukey-Kramer adjustment (P ≤ 0.05).

Use of 1.0 g of filamentous algae powder reduced the germination of amaranth by 22% from day 17 to day 22, and by 21% on average (P = 0.037). The other aquatic weeds tested had no effect on germination of terrestrial weeds at any treatment rate and at any time (Fig 5). In terms of biomass, the application of muskgrass powder resulted in a significant reduction on amaranth growth (P = 0.002); 0.1 g muskgrass reduced the biomass by 58%, 0.5 g by 55%, and 1.0 g by 68%. Use of 0.1 g and 1.0 g of water hyacinth powder reduced amaranth biomass by 50% and 44%, respectively (P = 0.011). However, the result obtained with 0.5 g was not different from the control. The same result was obtained with water lettuce: 0.1 g application resulted in 56% reduction, and 1.0 g in 53% reduction respectively (P = 0.026) while the biomass obtained after application of 0.5 g was not different from the biomass of the control. Both 0.5 g and 1.0 g of filamentous algae powder reduced biomass of amaranth significantly by 48% and 55%, respectively (P = 0.004). Hydrilla and duckweed powder showed no significant effect on amaranth growth at any rate (Fig 6).

**Fig 7:**
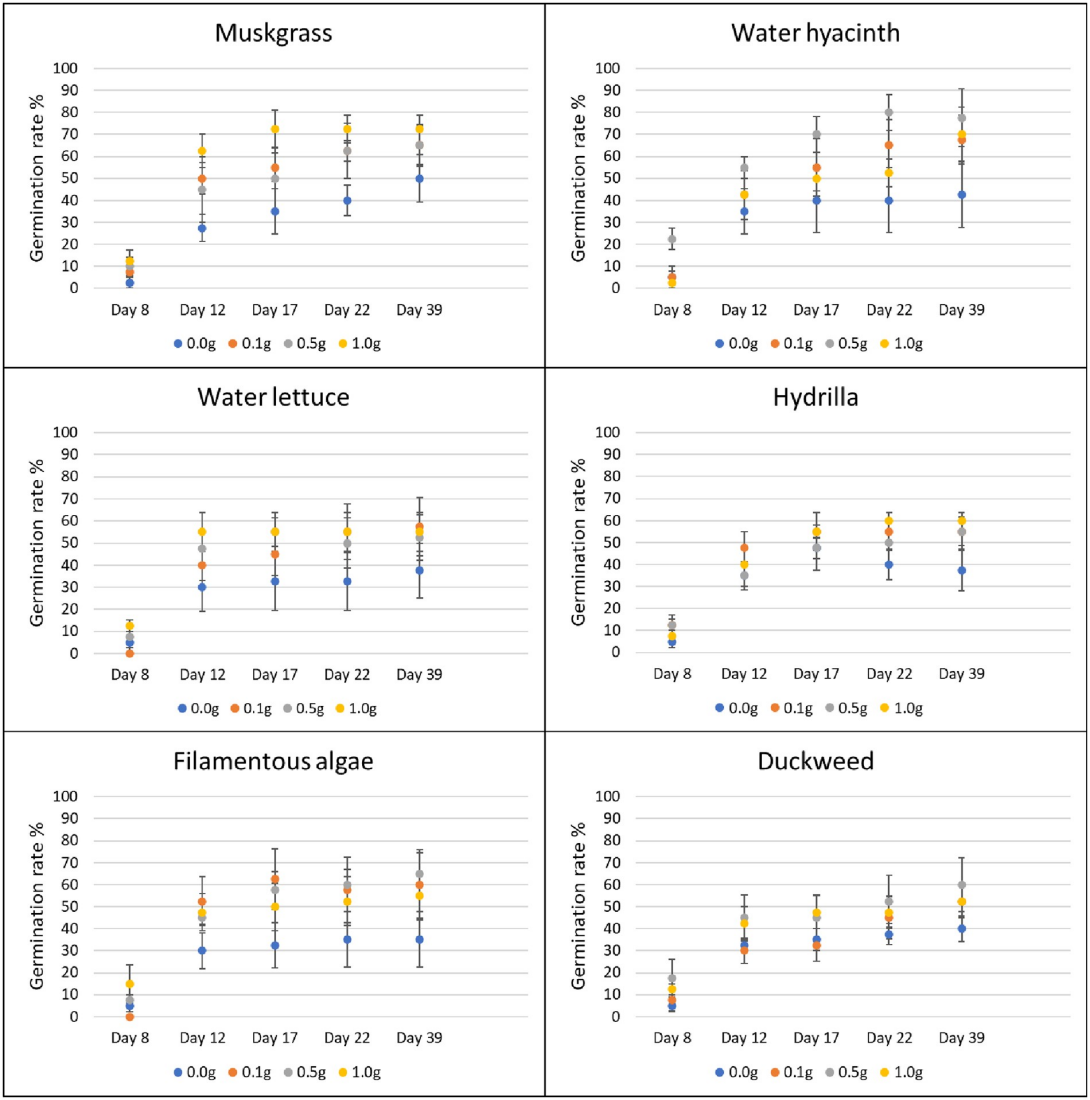
Effect of aquatic weed powder on germination rate of common ragweed. Vertical bars represent standard errors.

**Fig 8:**
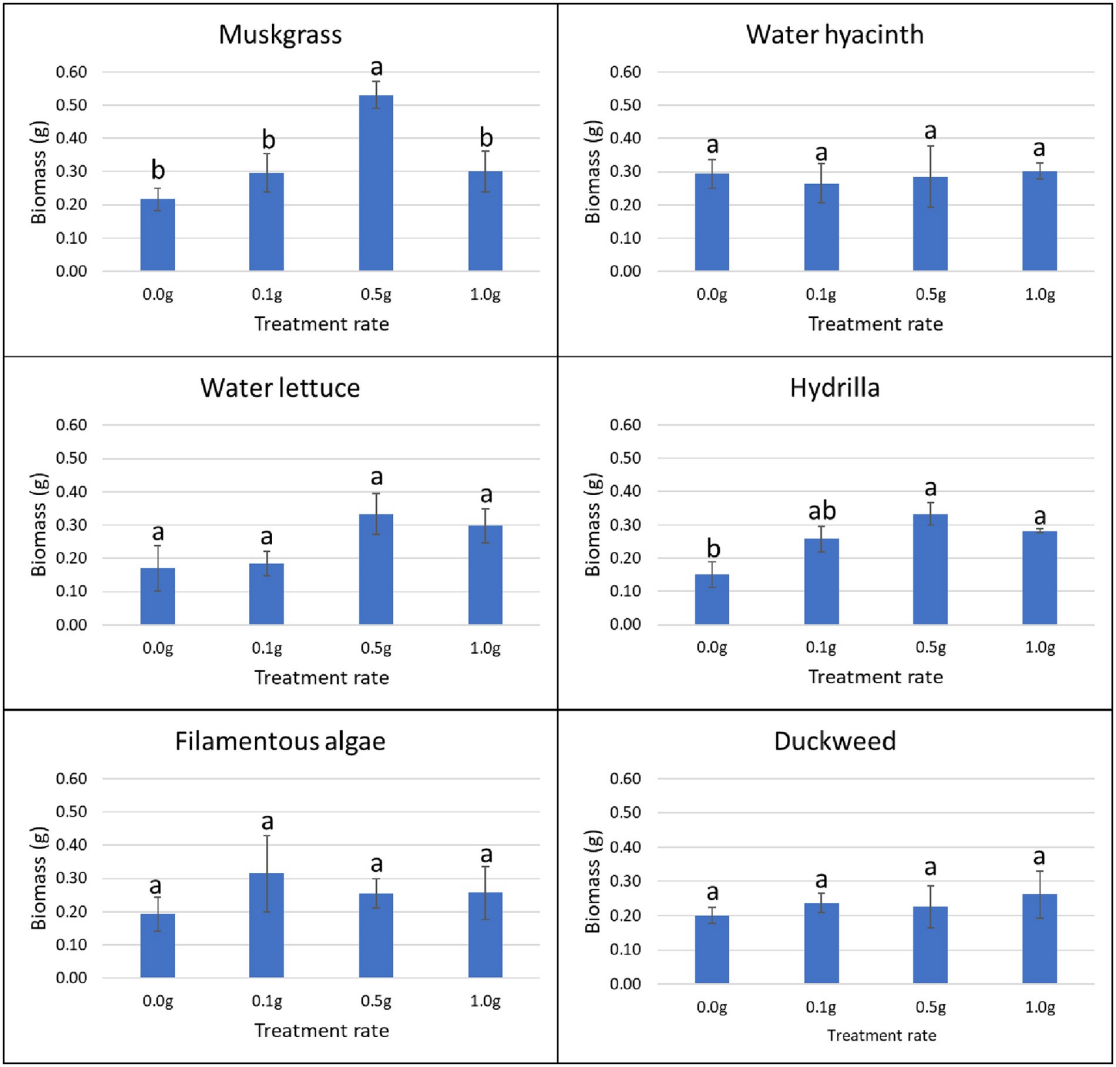
Effect of aquatic weed powder on biomass of common ragweed. Vertical bars represent standard errors of the mean. Bars designated by different lowercase letters are significantly different according to Tukey-Kramer adjustment (P ≤ 0.05).

For common ragweed, water hyacinth powder applied at 0.5 g increased germination by 350% on day 8 (P = 0.017). None of the other treatments showed any effects on common ragweed germination at any rate or time (Fig 7). Hydrilla improved the growth of common ragweed by 200% at 0.5 g and 87% at 1.0 g, respectively (P = 0.026). Muskgrass showed a 155% increase in common ragweed biomass at 0.5 g treatment rate (P = 0.005). None of the other aquatic weed powder had significant effects on the growth of common ragweed at any tested rate (Fig 8).

**Fig 9:**
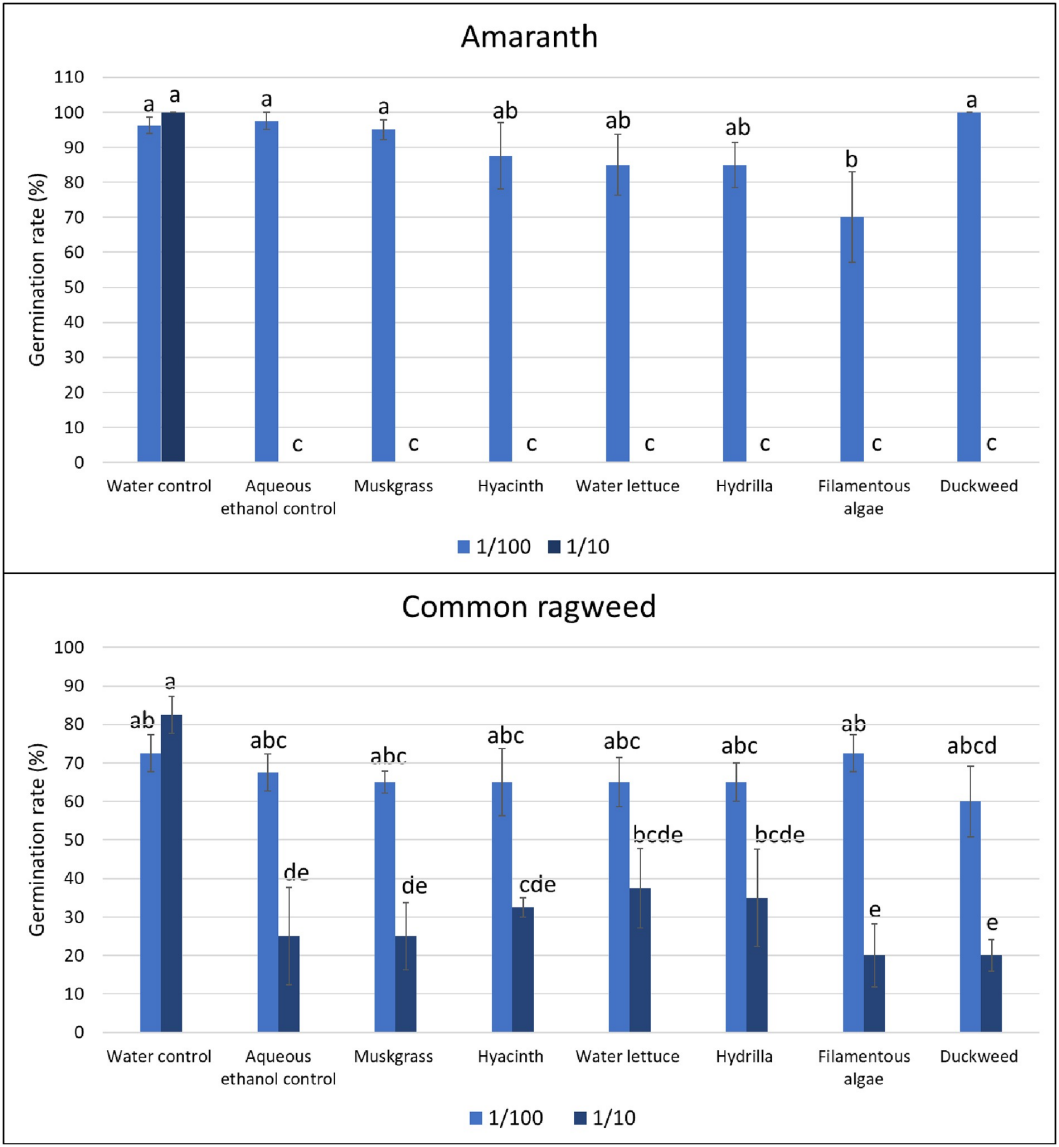
Effect of aquatic weed extracts on germination rate of amaranth and common ragweed. Vertical bars represent standard errors of the mean. Bars designated by different lowercase letters are significantly different according to Tukey-Kramer adjustment (P ≤ 0.05).

In the laboratory, the effect of aquatic weed extracts on seed germination of terrestrial weeds was observed. Filamentous algae extract at 1:100 dilution reduced 27% of the germination of amaranth seed compared to the water control and 28% to aqueous ethanol control (P < 0.001). Other treatment at 1:100 showed no significant difference in comparison to water control or aqueous ethanol control. All the treatments with 1/10 dilution, including the aqueous ethanol control showed no germination of amaranth. For common ragweed, none of the treatments showed a significant difference with the water control or aqueous ethanol control with 1:100 dilution. With 1:10 dilution, all the aquatic weed extracts showed a significant effect on germination rate compared with the water control, and the reduction in germination ranged from 54 to 76% (P < 0.001), but none of them showed difference with aqueous ethanol control (Fig 9). We were unable to make an observation on the effect of aqueous weed extracts on the germination of nutsedges as all the tubers failed to germinate in the Petri dishes.

The response of seed germination and growth of terrestrial weed species to six powdered aquatic weeds suggested that duckweed had an overall negative effect on nutsedge growth. Filamentous algae and muskgrass inhibited the germination and growth of amaranth, while water hyacinth and water lettuce have an inhibitory effect on the growth of amaranth.

These results are similar to the results by Bhadha et al., that the application of powdered water lettuce and filamentous algae demonstrated overall negative germination and root growth of plant species, including common lambsquarter, sorghum, snap bean and corn [11]. Mode of action of some allelochemicals can be similar to synthetic herbicides, and different allelochemicals may have different modes of action or physiological target sites [30]. Some allelochemicals, including flavonoids and fatty acids present in the aquatic weed powder, can act on different cellular processes, such as disrupting plasma membrane function and interrupting intracellular enzymes [11,31,32]. Besides directly targeting the weeds, the metabolites such as phenolics in the powder may release into the soil and alter its properties such as pH, organic matter, microorganisms, and nutrient status, resulting in a negative effect on plant germination and growth [33]. The results also showed no significant difference between 0.1 g to 1.0 g of duckweed on the growth of nutsedge, and also in terms of muskgrass on the growth of amaranth. This might due to that all the rates are at the same dose-response level of inhibitory effect on a nutsedge. For future work, higher application rates should be used to investigate their potential effect.

In contrast, common ragweed showed a positive response of germination with the application of powdered muskgrass and also of growth with powdered water hyacinth and hydrilla. This might suggest that the allelochemical in muskgrass and hydrilla had a stimulatory effect on the growth of common ragweed. The stimulatory effect of allelopathy was described in [34]; to show that the aqueous extract of mungbean (*Vigna radiata*) had a significant increase on seed germination and growth of plant species including sweet corn and okra [34]. The result might also suggest that common ragweed had high tolerance to the allelochemicals in these aquatic weeds and used the nutrients of powder for its own growth. Previous study had shown that aqueous extracts of the whole common ragweed plant inhibited seed germination of many plants including onion, oat, ryegrass, and Palmer amaranth [35]. Some allelochemicals such as phenolic acids and sesquiterpene-lactones were produced by ragweed in order to compete for nutrients and resist other allelochemicals [36,37].

Allelochemicals are known to induce hormesis in several plant species. Hormesis refers to biphasic dose-response with a low dose stimulatory or beneficial effect and a high dose inhibitory or toxic effect [38]. The current study showed that the lower rate (0.5 g) of hydrilla and muskgrass had a higher stimulatory effect on common ragweed than their higher rate (1.0 g) and suggested a hormetic response. A similar study also described the hormetic effect of aquatic weeds, that low application rates (0.03 g and 0.06 g) of water lettuce and filamentous algae promoted the root growth of corn, sorghum, and snap bean while high application rates (1.0 g) had an inhibitory effect on their root growth [11]. To further confirm the existence of hormesis, more application rates should be used in order to generate a more reliable dose-response model.

In the Petri dish experiment with aquatic weed extracts, aqueous ethanol control at 1/10 dilution showed no germination for amaranth, and nearly 70% less than the water control for ragweed. This suggested that the ethanol at 1/10 dilution had a potentially negative effect on seed germination of amaranth. Previous studies demonstrated that ethanol had different effects on the germination of different types of plant seeds. For instance, germination percentage of ryegrass (*Lolium*) seed decreased with ethanol concentration over 0.8% (v/v), while for bermudagrass (*Cynodon*), germination rate was increased with concentrations of ethanol from 0.1% to 3% (v/v) [39]. This also demonstrated that different concentrations of ethanol result in different effects on seed germination. However, filamentous algae at 1:100 dilution showed an inhibitory effect on seed germination of amaranth compared to aqueous ethanol control, which indicated the allelochemicals in the extract had an herbicidal effect on amaranth.

In this study, the presence of ethanol caused a significant reduction in seed germination of terrestrial weeds tested at 1/10 dilution, which made it hard to tell the activity of aquatic weed extracts at the same time. For future work, aqueous extraction could be used to avoid the impact of ethanol. In addition, other different types of solvents might be able to isolate the effective compounds. Previous studies described that aqueous methanol extract of duckweed was tested to have a strong inhibitory effect on root and shoot growth of terrestrial weeds including cress (*Lepidium sativum* L.), alfalfa (*Medicago sativa* L.), and timothy (*Phleum pratense* L.), and water lettuce aqueous methanol extract showed an inhibitory effect on shoot growth of cress and root growth of ryegrass (*Lolium multiforum* L.) and timothy [40]. Nutsedge tuber failed to germinate in the Petri dish, and this may be due to the lack of sufficient moisture for the tuber germination in the Petri dishes. Vermiculite could be a potential alternative medium for testing tuber germination instead of filter paper as it can hold relatively more moisture and is sterilizable.

## Conclusions

The objective of this study was to evaluate six aquatic weeds, including muskgrass (*Chara* spp.), water hyacinth (*Eichhornia crassipes*), water lettuce (*Pistia stratiotes*), hydrilla (*Hydrilla verticillate*), filamentous algae (*Lyngbya wollei*), and duckweed (*Lemna minor*), as potential biopesticides. The aquatic weed extracts were tested at dilutions 1:100 and 1:1,000 against 100 bacterial strains to evaluate their antimicrobial activity. Except for one bacterial isolate and one plant extract at the lowest dilution, the growth of these bacteria isolated from 25 plant and soil samples was not affected by the aquatic weed extracts in our testing conditions. The same aquatic weed extracts were used at 1:10 dilution to determine their insecticidal and antifeedant effects on the FAW. No significant treatment effect on FAW survival or growth was observed with the dip bioassay. However, using a diet incorporation bioassay allowed us to show that water lettuce extracts reduced the survival rate of FAW by 14%, and hydrilla and duckweed caused 11% and 9% average reduction of FAW growth, respectively. The aquatic weeds had no antifeedant effects on FAW. Four rates of powdered aquatic weeds and two dilutions of aquatic weed extracts (1:10 and 1:100) were used to determine their herbicidal effect towards seed germination and growth of nutsedge, amaranth, and common ragweed. In a greenhouse study, duckweed at all rates showed an inhibitory effect on the growth of nutsedge, ranging from 36% to 41%. Filamentous algae at 1.0 g reduced the germination rate of amaranth by 20% and biomass by 56%. Growth reductions of 68%, 49%, 53%, and 56% were observed in amaranth after application of muskgrass, water hyacinth, water lettuce, and filamentous algae, respectively. However, powdered muskgrass and hydrilla increased the growth of common ragweed by 200% and 145%, respectively. This suggested that common ragweed has a high tolerance to the allelochemicals present in the tested aquatic weeds. The powder used as a source of nutrient or the allelochemicals released from aquatic weed had a stimulatory effect on common ragweed. In the laboratory study, the highest reduction of seed germination (27–28%) was obtained for 1:100 dilution of filamentous algae extracts applied to amaranth. All the treatments at 1:10 dilution (including aqueous ethanol control) resulted in a significant reduction of seed germination of both amaranth and common ragweed. This indicated that the presence of ethanol inhibited seed germination, which interfered with allelochemicals present in the aquatic weed extracts.

This research contributed to pushing the boundary for future studies focused on reducing the dependence on synthetic pesticides and finding alternative strategies within the framework of promoting sustainable agriculture. Future studies could include identification of the chemicals contained in aquatic weed extracts in order to characterize the possible effective allelochemical compounds and their mode of action. Gas chromatography-mass spectrometry (GC-MS) analysis is a major analytical method to identify organic compounds in many studies. To further identify the molecules, the GC-MS spectrum of aquatic weed extracts could be compared with the known components stored in the National Institute Standard and Technology (NIST) library [29]. Since no universal extraction method exists for all allelochemicals, different solvents such as water, methanol, and acetone should also be evaluated in the future to identify the best extraction methods of allelochemicals from aquatic weeds.

## Supporting information

S1 Dataset Supplemental data. (DOCX)
