# Supplementary material for: Investigating the use of aquatic weeds as biopesticides towards promoting sustainable agriculture
Source: PLoS One. 2020 Aug 5;15(8):e0237258. doi: 10.1371/journal.pone.0237258 (PMC7406060; doi:10.1371/journal.pone.0237258)
Supplement: S1 Dataset — (DOCX) [file pone.0237258.s001.docx]

**S1 Dataset. Supplemental data.**

Table 1. Source and growth on different media of 100 bacterial isolates obtained from the Everglades agricultural area.

| No. | Source | Control | Aqueous ethanol | | Muskgrass | | Water hyacinth | | Water lettuce | | Hydrilla | | Filamentous algae | | | Duckweed | |
| --- | --- | --- | --- | --- | --- | --- | --- | --- | --- | --- | --- | --- | --- | --- | --- | --- | --- |
|  |  |  | 1/1000 | 1/100 | 1/1000 | 1/100 | 1/1000 | 1/100 | 1/1000 | 1/100 | 1/1000 | 1/100 | 1/1000 | 1/100 | 1/1000 | | 1/100 |
| 1 | St Augustine grass | + | + | + | + | + | + | + | + | + | + | + | + | + | + | | + |
| 2 | St Augustine grass | + | + | + | + | + | + | + | + | + | + | + | + | + | + | | + |
| 3 | St Augustine grass | + | + | + | + | + | + | + | + | + | + | + | + | + | + | | + |
| 4 | Sweet corn leaf | + | + | + | + | + | + | + | + | + | + | + | + | + | + | | + |
| 5 | Sweet corn leaf | + | + | + | + | + | + | + | + | + | + | + | + | + | + | | + |
| 6 | Sweet corn leaf | + | + | + | + | + | + | + | + | + | + | + | + | + | + | | + |
| 7 | Sweet corn leaf | + | + | + | + | + | + | + | + | + | + | + | + | + | + | | + |
| 8 | Sweet corn leaf | + | + | + | + | + | + | + | + | + | + | + | + | + | + | | + |
| 9 | Sweet corn leaf | + | + | + | + | + | + | + | + | + | + | + | + | + | + | | + |
| 10 | Sweet corn leaf | + | + | + | + | + | + | + | + | + | + | + | + | + | + | | + |
| 11 | Papaya tree leaf | + | + | + | + | -w | + | + | + | + | + | + | + | + | + | | + |
| 12 | Melaleuca viminalis | + | + | + | + | + | + | + | + | + | + | + | + | + | + | | + |
| 13 | Papaya tree leaf | + | + | + | + | + | + | + | + | + | + | + | + | + | + | | + |
| 14 | Papaya tree leaf | + | + | + | + | + | + | + | + | + | + | + | + | + | + | | + |

Table 1. Continued

| No. | Source | Control | Aqueous ethanol | | Muskgrass | | Water hyacinth | | Water lettuce | | Hydrilla | | Filamentous algae | | Duckweed | |
| --- | --- | --- | --- | --- | --- | --- | --- | --- | --- | --- | --- | --- | --- | --- | --- | --- |
|  |  |  | 1/1000 | 1/100 | 1/1000 | 1/100 | 1/1000 | 1/100 | 1/1000 | 1/100 | 1/1000 | 1/100 | 1/1000 | 1/100 | 1/1000 | 1/100 |
| 15 | Melaleuca viminalis | + | + | + | + | + | + | + | + | + | + | + | + | + | + | + |
| 16 | Papaya tree leaf | + | + | + | + | + | + | + | + | + | + | + | + | + | + | + |
| 17 | Papaya tree leaf | + | + | + | + | + | + | + | + | + | + | + | + | + | + | + |
| 18 | Papaya tree leaf | + | + | + | + | + | + | + | + | + | + | + | + | + | + | + |
| 19 | Papaya tree leaf | + | + | + | + | + | + | + | + | + | + | + | + | + | + | + |
| 20 | Palm tree leaf | + | + | + | + | + | + | + | + | + | + | + | + | + | + | + |
| 21 | Palm tree leaf | + | + | + | + | + | + | + | + | + | + | + | + | + | + | + |
| 22 | Palm tree leaf | + | + | + | + | + | + | + | + | + | + | + | + | + | + | + |
| 23 | Melaleuca viminalis | + | + | + | + | + | + | + | + | + | + | + | + | + | + | + |
| 24 | St Augustine grass | + | + | + | + | + | + | + | + | + | + | + | + | + | + | + |
| 25 | Papaya tree leaf | + | + | + | + | + | + | + | + | + | + | + | + | + | + | + |
| 26 | Melaleuca viminalis | + | + | + | + | + | + | + | + | + | + | + | + | + | + | + |
| 27 | Rice upper leaf | + | + | + | + | + | + | + | + | + | + | + | + | + | + | + |
| 28 | Rice upper leaf | + | + | + | + | + | + | + | + | + | + | + | + | + | + | + |
| 29 | Melaleuca viminalis | + | + | + | + | + | + | + | + | + | + | + | + | + | + | + |

Table 1. Continued

| No. | Source | Control | Aqueous ethanol | | Muskgrass | | Water hyacinth | | Water lettuce | | Hydrilla | | Filamentous algae | | Duckweed | |
| --- | --- | --- | --- | --- | --- | --- | --- | --- | --- | --- | --- | --- | --- | --- | --- | --- |
|  |  |  | 1/1000 | 1/100 | 1/1000 | 1/100 | 1/1000 | 1/100 | 1/1000 | 1/100 | 1/1000 | 1/100 | 1/1000 | 1/100 | 1/1000 | 1/100 |
| 30 | Rice upper leaf | + | + | + | + | + | + | + | + | + | + | + | + | + | + | + |
| 31 | Rice upper leaf | + | + | + | + | + | + | + | + | + | + | + | + | + | + | + |
| 32 | Rice upper leaf | + | + | + | + | + | + | + | + | + | + | + | + | + | + | + |
| 33 | Rice lower leaf | + | + | + | + | + | + | + | + | + | + | + | + | + | + | + |
| 34 | Rice lower leaf | + | + | + | + | + | + | + | + | + | + | + | + | + | + | + |
| 35 | Rice lower leaf | + | + | + | + | + | + | + | + | + | + | + | + | + | + | + |
| 36 | Rice lower leaf | + | + | + | + | + | + | + | + | + | + | + | + | + | + | + |
| 37 | Rice lower leaf | + | + | + | + | + | + | + | + | + | + | + | + | + | + | + |
| 38 | Rice lower leaf | + | + | + | + | + | + | + | + | + | + | + | + | + | + | + |
| 39 | Sugarcane CV1040 | + | + | + | + | + | + | + | + | + | + | + | + | + | + | + |
| 40 | Sugarcane CV1041 | + | + | + | + | + | + | + | + | + | + | + | + | + | + | + |
| 41 | Sugarcane CV1042 | + | + | + | + | + | + | + | + | + | + | + | + | + | + | + |
| 42 | Sugarcane CV1043 | + | + | + | + | + | + | + | + | + | + | + | + | + | + | + |
| 43 | Sugarcane CV1044 | + | + | + | + | + | + | + | + | + | + | + | + | + | + | + |
| 44 | Sugarcane CV4725 | + | + | + | + | + | + | + | + | + | + | + | + | + | + | + |

Table 1. Continued

| No. | Source | Control | Aqueous ethanol | | Muskgrass | | Water hyacinth | | Water lettuce | | Hydrilla | | Filamentous algae | | | Duckweed | | |
| --- | --- | --- | --- | --- | --- | --- | --- | --- | --- | --- | --- | --- | --- | --- | --- | --- | --- | --- |
|  |  |  | 1/1000 | 1/100 | 1/1000 | 1/100 | 1/1000 | 1/100 | 1/1000 | 1/100 | 1/1000 | 1/100 | 1/1000 | 1/100 | 1/1000 | | 1/100 |  |
| 45 | Melaleuca viminalis | + | + | + | + | + | + | + | + | + | + | + | + | + | + | | + |  |
| 46 | Sugarcane CV4727 | + | + | + | + | + | + | + | + | + | + | + | + | + | + | | + |  |
| 47 | Sugarcane CV4728 | + | + | + | + | + | + | + | + | + | + | + | + | + | + | | + |  |
| 48 | Melaleuca viminalis | + | + | + | + | + | + | + | + | + | + | + | + | + | + | | + |  |
| 49 | Cypress tree | + | + | + | + | + | + | + | + | + | + | + | + | + | + | | + |  |
| 50 | Cypress tree | + | + | + | + | + | + | + | + | + | + | + | + | + | + | | + |  |
| 51 | Sorghum Bicolor | + | + | + | + | + | + | + | + | + | + | + | + | + | + | | + |  |
| 52 | Sorghum Bicolor | + | + | + | + | + | + | + | + | + | + | + | + | + | + | | + |  |
| 53 | Sorghum Bicolor | + | + | + | + | + | + | + | + | + | + | + | + | + | + | | + |  |
| 54 | Sorghum Bicolor | + | + | + | + | + | + | + | + | + | + | + | + | + | + | | + |  |
| 55 | Sorghum Bicolor | + | + | + | + | + | + | + | + | + | + | + | + | + | + | | + |  |
| 56 | Sorghum Bicolor Aphid | + | + | + | + | + | + | + | + | + | + | + | + | + | + | | + |  |
| 57 | Sorghum Bicolor Aphid | + | + | + | + | + | + | + | + | + | + | + | + | + | + | | + |  |
| 58 | Sorghum Bicolor Aphid | + | + | + | + | + | + | -w | + | + | + | + | + | -w | + | | + |  |
| 59 | Sorghum Bicolor Aphid | + | + | + | + | + | + | + | + | + | + | + | + | + | + | | + |  |

Table 1. Continued

| No. | Source | Control | Aqueous ethanol | | Muskgrass | | Water hyacinth | | Water lettuce | | Hydrilla | | Filamentous algae | | Duckweed | |
| --- | --- | --- | --- | --- | --- | --- | --- | --- | --- | --- | --- | --- | --- | --- | --- | --- |
|  |  |  | 1/1000 | 1/100 | 1/1000 | 1/100 | 1/1000 | 1/100 | 1/1000 | 1/100 | 1/1000 | 1/100 | 1/1000 | 1/100 | 1/1000 | 1/100 |
| 60 | Sorghum Bicolor Aphid | + | + | + | + | + | + | + | + | + | + | + | + | + | + | + |
| 61 | Melaleuca viminalis | + | + | + | + | + | + | + | + | + | + | + | + | + | + | + |
| 62 | Sorghum Almum | + | + | + | + | + | + | + | + | + | + | + | + | + | + | + |
| 63 | Sorghum Almum | + | + | + | + | + | + | + | + | + | + | + | + | + | + | + |
| 64 | Sorghum Almum | + | + | + | + | + | + | + | + | + | + | + | + | + | + | + |
| 65 | Sorghum Almum | + | + | + | + | + | + | + | + | + | + | + | + | + | + | + |
| 66 | Sorghum Almum | + | + | + | + | + | + | + | + | + | + | + | + | + | + | + |
| 67 | Sorghum Almum Aphid | + | + | + | + | + | + | + | + | + | + | + | + | + | + | + |
| 68 | Sorghum Almum Aphid | + | + | + | + | + | + | + | + | + | + | + | + | + | + | + |
| 69 | Sorghum Almum Aphid | + | + | + | + | + | + | + | + | + | + | + | + | + | + | + |
| 70 | Melaleuca viminalis | + | + | + | + | + | + | + | + | + | + | + | + | + | + | + |
| 71 | Sugarcane soil | + | + | + | + | + | + | + | + | + | + | + | + | + | + | + |
| 72 | Grain soil | + | + | + | + | -w | + | + | + | + | + | + | + | + | + | + |
| 73 | Grain soil | + | + | + | + | + | + | + | + | + | + | + | + | + | + | + |
| 74 | Grain soil | + | + | + | + | + | + | + | + | + | + | + | + | + | + | + |

Table 1. Continued

| No. | Source | Control | Aqueous ethanol | | Muskgrass | | Water hyacinth | | Water lettuce | | Hydrilla | | Filamentous algae | | | Duckweed | | |
| --- | --- | --- | --- | --- | --- | --- | --- | --- | --- | --- | --- | --- | --- | --- | --- | --- | --- | --- |
|  |  |  | 1/1000 | 1/100 | 1/1000 | 1/100 | 1/1000 | 1/100 | 1/1000 | 1/100 | 1/1000 | 1/100 | 1/1000 | 1/100 | 1/1000 | | 1/100 |  |
| 75 | Pink flower | + | + | + | + | + | + | + | + | + | + | + | + | + | + | | + |  |
| 76 | Pink flower | + | + | + | + | + | + | + | + | + | + | + | + | + | + | | + |  |
| 77 | Pink flower | + | + | + | + | + | + | + | + | + | + | + | + | + | + | | + |  |
| 78 | White flower | + | + | + | + | + | + | + | + | + | + | + | + | + | + | | + |  |
| 79 | Red flower | + | + | + | + | + | + | + | + | + | + | + | + | + | + | | + |  |
| 80 | Red flower | + | + | + | + | + | + | + | + | + | + | + | + | + | + | | + |  |
| 81 | Red flower | + | + | + | + | + | + | + | + | + | + | + | + | + | + | | + |  |
| 82 | Gainesville soil | + | + | + | + | + | + | + | + | + | + | + | + | + | + | | + |  |
| 83 | St Agustine grass soil | + | + | + | + | + | + | + | + | + | + | + | + | + | + | | + |  |
| 84 | St Agustine grass soil | + | + | + | + | + | + | + | + | + | + | + | + | + | + | | + |  |
| 85 | St Agustine grass soil | + | + | + | + | + | + | + | + | + | + | + | + | + | + | | + |  |
| 86 | St Agustine grass soil | + | + | + | + | + | + | + | + | + | + | + | + | + | + | | + |  |
| 87 | St Agustine grass soil | + | + | + | + | + | + | + | + | + | + | + | + | + | + | | + |  |
| 88 | Milton soil | + | + | + | + | + | + | + | + | + | + | + | + | + | + | | + |  |
| 89 | Homestead soil | + | + | + | + | + | + | + | + | + | + | + | + | + | + | | + |  |

Table 1. Continued

| No. | Source | Control | Aqueous ethanol | | Muskgrass | | Water hyacinth | | Water lettuce | | Hydrilla | | Filamentous algae | | Duckweed | |
| --- | --- | --- | --- | --- | --- | --- | --- | --- | --- | --- | --- | --- | --- | --- | --- | --- |
|  |  |  | 1/1000 | 1/100 | 1/1000 | 1/100 | 1/1000 | 1/100 | 1/1000 | 1/100 | 1/1000 | 1/100 | 1/1000 | 1/100 | 1/1000 | 1/100 |
| 90 | Homestead soil | + | + | + | + | + | + | + | + | + | + | + | + | + | + | + |
| 91 | Lettuce leaf | + | + | + | + | + | + | + | + | + | + | + | + | + | + | + |
| 92 | Lettuce leaf | + | + | + | + | + | + | + | + | + | + | + | + | + | + | + |
| 93 | Lettuce leaf | + | + | + | + | + | + | + | + | + | + | + | + | + | + | + |
| 94 | Red lettuce leaf | + | + | + | + | + | + | + | + | + | + | + | + | + | + | + |
| 95 | Red lettuce leaf | + | + | + | + | + | + | + | + | + | + | + | + | + | + | + |
| 96 | Red lettuce leaf | + | + | + | + | + | + | + | + | + | + | + | + | + | + | + |
| 97 | Red lettuce leaf | + | + | + | + | + | + | + | + | + | + | + | + | + | + | + |
| 98 | Red lettuce leaf | + | + | + | + | + | + | + | + | + | + | + | + | + | + | + |
| 99 | Ft Pierce soil | + | + | + | + | + | + | + | + | + | + | + | + | + | + | + |
| 100 | Ft Pierce soil | + | + | + | + | + | + | + | + | + | + | + | + | + | + | + |

+ = bacterial growth; -w = partial bacterial growth; Control = basal medium (BM); Aqueous ethanol = BM supplemented without extraction solvent; For each aquatic weed: BM supplemented with two different dilutions of plant extract (1/100 and 1/1000).

Table 2. Effect of powdered aquatic weed on germination rate and biomass of nutsedge, negative value means the increase of the biomass.

| Effect of muskgrass powder on germination and biomass of nutsedge | | | | | | | | | |
| --- | --- | --- | --- | --- | --- | --- | --- | --- | --- |
| Rate (g) | Day 8 | Day 12 | Day 17 | Day 22 | Day 39 | Average | Biomass (g) | Biomass reduction (%) |  |
| 0.0 | 0.0 | 50.0±10.0a | 80.0±4.1a | 85.0±2.9a | 90.0±4.1a | 61.0±3.4a | 4.8±0.5a |  |  |
| 0.1 | 0.0 | 50.0±4.1a | 77.5±7.5a | 90.0±4.1a | 95.0±2.9a | 62.5±2.1a | 4.8±0.4a | 0.7 |  |
| 0.5 | 0.0 | 57.5±9.5a | 85.0±2.9a | 95.0±2.9a | 95.0±2.9a | 66.5±2.2a | 3.9±0.3a | 19.8 |  |
| 1.0 | 0.0 | 52.5±10.3a | 75.0±8.7a | 85.0±6.5a | 90.0±4.1a | 60.5±5.6a | 4.2±0.2a | 12.0 |  |
| P-value |  | 0.9271 | 0.5827 | 0.3272 | 0.3641 | 0.6250 | 0.2990 |  |  |

| Effect of water hyacinth powder on germination and biomass of nutsedge | | | | | | | | | |
| --- | --- | --- | --- | --- | --- | --- | --- | --- | --- |
| Rate (g) | Day 8 | Day 12 | Day 17 | Day 22 | Day 39 | Average | Biomass (g) | Biomass reduction (%) |  |
| 0.0 | 0.0 | 52.5±9.5a | 67.5±9.5a | 80.0±8.2a | 80.0±8.2a | 56.0±6.0a | 4.9±0.5a |  |  |
| 0.1 | 0.0 | 57.5±4.8a | 67.5±4.8a | 82.5±7.5a | 82.5±7.5a | 58.0±4.3a | 4.2±0.3a | 13.8 |  |
| 0.5 | 0.0 | 62.5±8.5a | 80.0±4.1a | 82.5±2.5a | 85.0±2.9a | 62.0±3.2a | 4.4±0.4a | 9.1 |  |
| 1.0 | 0.0 | 47.5±6.3a | 77.5±11.1a | 95.0±2.9a | 95.0±2.9a | 63.0±4.1a | 4.3±0.5a | 13.1 |  |
| P-value |  | 0.5296 | 0.6044 | 0.2797 | 0.3669 | 0.7057 | 0.6163 |  |  |

Table 2. Continued

| Effect of water lettuce powder on germination and biomass of nutsedge | | | | | | | | | |
| --- | --- | --- | --- | --- | --- | --- | --- | --- | --- |
| Rate (g) | Day 8 | Day 12 | Day 17 | Day 22 | Day 39 | Average | Biomass (g) | Biomass reduction (%) |  |
| 0.0 | 0.0 | 50.0±5.8a | 67.5±4.8a | 87.5±4.8a | 92.5±2.5a | 59.5±0.5a | 4.3±0.6a |  |  |
| 0.1 | 0.0 | 47.5±10.3a | 62.5±12.5a | 77.5±2.5a | 82.5±4.8a | 54.0±5.0a | 3.5±0.6a | 18.7 |  |
| 0.5 | 0.0 | 55.0±5.0a | 77.5±4.8a | 85.0±6.5a | 87.5±7.5a | 61.0±3.4a | 5.2±0.3a | -20.0 |  |
| 1.0 | 0.0 | 55.0±6.5a | 72.5±4.8a | 80.0±4.1a | 80.0±4.1a | 57.5±3.2a | 3.7±0.3a | 15.6 |  |
| P-value |  | 0.5664 | 0.5851 | 0.5493 | 0.3986 | 0.4088 | 0.1198 |  |  |

| Effect of hydrilla powder on germination and biomass of nutsedge | | | | | | | | | |
| --- | --- | --- | --- | --- | --- | --- | --- | --- | --- |
| Rate (g) | Day 8 | Day 12 | Day 17 | Day 22 | Day 39 | Average | Biomass (g) | Biomass reduction  (%) |  |
| 0.0 | 0.0 | 50.0±7.1a | 70.0±11.5a | 85.0±9.6a | 87.5±9.5a | 58.5±6.4a | 4.2±0.4a |  |  |
| 0.1 | 0.0 | 40.0±10.1a | 60.0±10.8a | 82.5±8.5a | 85.0±6.5a | 53.5±7.1a | 3.9±0.5a | 6.3 |  |
| 0.5 | 0.0 | 42.5±6.3a | 67.5±6.3a | 80.0±8.2a | 80.0±8.2a | 54.0±5.7a | 5.4±0.6a | -28.8 |  |
| 1.0 | 0.0 | 57.5±2.5a | 75.0±6.5a | 82.5±4.8a | 87.5±2.5a | 60.5±2.8a | 3.9±0.6a | 6.7 |  |
| P-value |  | 0.1912 | 0.5012 | 0.9580 | 0.8594 | 0.6457 | 0.1736 |  |  |

Table 2. Continued

| Effect of filamentous algae powder on germination and biomass of nutsedge | | | | | | | | | |
| --- | --- | --- | --- | --- | --- | --- | --- | --- | --- |
| Rate (g) | Day 8 | Day 12 | Day 17 | Day 22 | Day 39 | Average | Biomass (g) | Biomass reduction  (%) |  |
| 0.0 | 0.0 | 40.0±5.8a | 62.5±12.5a | 85.0±6.5a | 95.0±5.0a | 56.5±4.3a | 4.0±0.2a |  |  |
| 0.1 | 0.0 | 45.0±5.0a | 55.0±8.7a | 67.5±11.1a | 72.5±11.1a | 48.0±7.0a | 4.7±0.8a | -15.4 |  |
| 0.5 | 0.0 | 42.5±9.5a | 65.0±9.6a | 70.0±9.1a | 72.5±7.5a | 50.0±5.8a | 3.4±0.3a | 15.2 |  |
| 1.0 | 0.0 | 52.5±2.5a | 65.0±2.9a | 80.0±4.1a | 80.0±4.1a | 55.5±2.2a | 4.5±0.4a | -10.3 |  |
| P-value |  | 0.5753 | 0.8182 | 0.3936 | 0.1883 | 0.5808 | 0.3795 |  |  |

| Effect of duckweed powder on germination and biomass of nutsedge | | | | | | | | | |
| --- | --- | --- | --- | --- | --- | --- | --- | --- | --- |
| Rate (g) | Day 8 | Day 12 | Day 17 | Day 22 | Day 39 | Average | Biomass (g) | Biomass reduction (%) |  |
| 0.0 | 0.0 | 60.0±10.0a | 75.0±2.9a | 90.0±4.1a | 92.5±4.8a | 63.5±3.4a | 6.0±0.6a |  |  |
| 0.1 | 0.0 | 42.5±4.8a | 57.5±11.1a | 70.0±9.1a | 77.5±10.3a | 49.5±6.9a | 3.8±0.5b | 36.6 |  |
| 0.5 | 0.0 | 50.0±8.2a | 72.5±4.8a | 77.5±4.8a | 80.0±4.1a | 56.0±3.8a | 3.6±0.2b | 40.5 |  |
| 1.0 | 0.0 | 40.0±12.9a | 65.0±9.6a | 67.5±6.3a | 72.5±4.8a | 49.0±6.0a | 3.8±0.7b | 36.3 |  |
| P-value |  | 0.3280 | 0.4182 | 0.1028 | 0.1512 | 0.2013 | 0.0416 |  |  |

Table 3. Effect of powdered aquatic weed on germination rate and biomass of amaranth, negative value means the increase of the biomass.

| Effect of muskgrass powder on germination and biomass of amaranth | | | | | | | | | |
| --- | --- | --- | --- | --- | --- | --- | --- | --- | --- |
| Rate (g) | Day 8 | Day 12 | Day 17 | Day 22 | Day 39 | Average | Biomass (g) | Biomass reduction (%) |  |
| 0.0 | 80.0a | 85.0±2.9a | 90.0±4.1a | 92.5±4.8a | 90.0±4.1a | 87.5±1.9 a | 0.27±0.03 a |  |  |
| 0.1 | 72.5±21.0a | 77.5±19.3a | 77.5±19.3a | 77.5±19.3a | 77.5±19.3a | 76.5±19.6a | 0.12±0.03 b | 57.6 |  |
| 0.5 | 75.0±6.5a | 77.5±4.8a | 87.5±6.3a | 90.0±7.1a | 90.0±7.1a | 84.0±6.1a | 0.12±0.004 b | 55.4 |  |
| 1.0 | 92.5±2.5a | 95.0±2.9a | 95.0±2.9a | 95.0±2.9a | 90.0±4.1a | 93.5±2.1a | 0.09±0.02 b | 67.9 |  |
| P-value | 0.5285 | 0.5157 | 0.5419 | 0.4796 | 0.7438 | 0.6030 | 0.0018 |  |  |

| Effect of water hyacinth powder on germination and biomass of amaranth | | | | | | | | | |
| --- | --- | --- | --- | --- | --- | --- | --- | --- | --- |
| Rate (g) | Day 8 | Day 12 | Day 17 | Day 22 | Day 39 | Average | Biomass (g) | Biomass reduction (%) |  |
| 0.0 | 80.0±4.1a | 87.5±2.5a | 87.5±2.5a | 87.5±2.5a | 85.0±2.9a | 85.5±2.2a | 0.19±0.02 |  |  |
| 0.1 | 82.5±11.8a | 87.5±9.5a | 85.0±7.1a | 90.0±7.1a | 85.0±6.5a | 87.0±8.1a | 0.10±0.01b | 49.5 |  |
| 0.5 | 72.5±2.5a | 82.5±2.5a | 85.0±2.9a | 80.0±4.1a | 75.0±2.9a | 79.0±1.9a | 0.14±0.02ab | 26.8 |  |
| 1.0 | 82.5±6.3a | 82.5±6.3a | 90.0±5.0a | 87.5±4.8a | 80.0±4.1a | 83.5±4.3a | 0.11±0.01b | 44.2 |  |
| P-value | 0.7484 | 0.8663 | 0.8302 | 0.5978 | 0.2959 | 0.6784 | 0.0109 |  |  |

Table 3. Continued

| Effect of water lettuce powder on germination and biomass of amaranth | | | | | | | | | |
| --- | --- | --- | --- | --- | --- | --- | --- | --- | --- |
| Rate (g) | Day 8 | Day 12 | Day 17 | Day 22 | Day 39 | Average | Biomass (g) | Biomass reduction (%) |  |
| 0.0 | 90.0±5.8a | 92.5±4.8a | 92.5±4.8a | 92.5±4.8a | 97.5±2.5a | 93.0±4.4a | 0.18±0.03a |  |  |
| 0.1 | 77.5±4.8a | 82.5±4.8a | 82.5±4.8a | 82.5±4.8a | 80.0±4.1a | 81.0±4.0a | 0.08±0.003b | 55.6 |  |
| 0.5 | 75.0±5.0a | 85.0±6.5a | 85.0±6.5a | 87.5±4.8a | 80.0±7.1a | 82.5±5.6a | 0.12±0.02ab | 30.9 |  |
| 1.0 | 77.5±10.3a | 82.5±10.3a | 82.5±10.3a | 80.0±9.1a | 80.0±9.1a | 80.5±9.7a | 0.08±0.01b | 52.8 |  |
| P-value | 0.3272 | 0.7337 | 0.7337 | 0.5167 | 0.2432 | 0.4990 | 0.0262 |  |  |

| Effect of hydrilla powder on germination and biomass of amaranth | | | | | | | | | |
| --- | --- | --- | --- | --- | --- | --- | --- | --- | --- |
| Rate (g) | Day 8 | Day 12 | Day 17 | Day 22 | Day 39 | Average | Biomass (g) | Biomass reduction (%) |  |
| 0.0 | 90.0±4.1a | 95.0±2.9a | 95.0±2.9a | 95.0±2.9a | 95.0±2.9a | 94.0±2.9a | 0.13±0.01a |  |  |
| 0.1 | 77.5±8.5a | 85.0±5.0a | 92.5±4.8a | 92.5±2.5a | 95.0±2.9a | 88.5±4.0a | 0.17±0.02a | -24.6 |  |
| 0.5 | 77.5±2.5a | 85.0±5.0a | 87.5±4.8a | 87.5±4.8a | 85.0±2.9a | 84.5±3.6a | 0.18±0.02a | -30.6 |  |
| 1.0 | 85.0±6.5a | 92.5±4.8a | 92.5±4.8a | 92.5±2.5a | 82.5±4.8a | 89.0±3.5a | 0.14±0.02a | -3.7 |  |
| P-value | 0.4212 | 0.2019 | 0.4885 | 0.3763 | 0.1011 | 0.2717 | 0.3988 |  |  |

Table 3. Continued

| Effect of filamentous algae powder on germination and biomass of nutsedge | | | | | | | | | |
| --- | --- | --- | --- | --- | --- | --- | --- | --- | --- |
| Rate (g) | Day 8 | Day 12 | Day 17 | Day 22 | Day 39 | Average | Biomass (g) | Biomass reduction (%) |  |
| 0.0 | 0.0 | 40.0±5.8a | 62.5±12.5a | 85.0±6.5a | 95.0±5.0a | 56.5±4.3a | 4.0±0.2a |  |  |
| 0.1 | 0.0 | 45.0±5.0a | 55.0±8.7a | 67.5±11.1a | 72.5±11.1a | 48.0±7.0a | 4.7±0.8a | -15.4 |  |
| 0.5 | 0.0 | 42.5±9.5a | 65.0±9.6a | 70.0±9.1a | 72.5±7.5a | 50.0±5.8a | 3.4±0.3a | 15.2 |  |
| 1.0 | 0.0 | 52.5±2.5a | 65.0±2.9a | 80.0±4.1a | 80.0±4.1a | 55.5±2.2a | 4.5±0.4a | -10.3 |  |
| P-value |  | 0.5753 | 0.8182 | 0.3936 | 0.1883 | 0.5808 | 0.3795 |  |  |

| Effect of duckweed powder on germination and biomass of nutsedge | | | | | | | | | |
| --- | --- | --- | --- | --- | --- | --- | --- | --- | --- |
| Rate (g) | Day 8 | Day 12 | Day 17 | Day 22 | Day 39 | Average | Biomass (g) | Biomass reduction (%) |  |
| 0.0 | 0.0 | 60.0±10.0a | 75.0±2.9a | 90.0±4.1a | 92.5±4.8a | 63.5±3.4a | 6.0±0.6a |  |  |
| 0.1 | 0.0 | 42.5±4.8a | 57.5±11.1a | 70.0±9.1a | 77.5±10.3a | 49.5±6.9a | 3.8±0.5b | 36.6 |  |
| 0.5 | 0.0 | 50.0±8.2a | 72.5±4.8a | 77.5±4.8a | 80.0±4.1a | 56.0±3.8a | 3.6±0.2b | 40.5 |  |
| 1.0 | 0.0 | 40.0±12.9a | 65.0±9.6a | 67.5±6.3a | 72.5±4.8a | 49.0±6.0a | 3.8±0.7b | 36.3 |  |
| P-value |  | 0.3280 | 0.4182 | 0.1028 | 0.1512 | 0.2013 | 0.0416 |  |  |

Table 4. Effect of powdered aquatic weed on germination rate and biomass of common ragweed, negative value means the increase of the biomass.

| Effect of muskgrass powder on germination and biomass of common ragweed | | | | | | | | | |
| --- | --- | --- | --- | --- | --- | --- | --- | --- | --- |
| Rate (g) | Day 8 | Day 12 | Day 17 | Day 22 | Day 39 | Average | Biomass (g) | Biomass reduction (%) |  |
| 0.0 | 2.5±2.5a | 27.5±6.3a | 35.0±10.4a | 40.0±7.1a | 50.0±10.8a | 31.0±6.4a | 0.22±0.03b |  |  |
| 0.1 | 7.5±4.8a | 50.0±7.1a | 55.0±6.5a | 62.5±4.8a | 65.0±8.7a | 48.0±5.0a | 0.30±0.06b | -36.4 |  |
| 0.5 | 10.0±4.1a | 45.0±15.0a | 50.0±14.1a | 62.5±12.5a | 65.0±9.6a | 46.5±9.8a | 0.53±0.04a | -144.7 |  |
| 1.0 | 12.5±4.8a | 62.5±7.5a | 72.5±8.5a | 72.5±6.3a | 72.5±6.3a | 58.5±5.8a | 0.30±0.06b | -38.3 |  |
| P-value | 0.3707 | 0.1953 | 0.1097 | 0.1030 | 0.3972 | 0.1194 | 0.0046 |  |  |

| Effect of water hyacinth powder on germination and biomass of common ragweed | | | | | | | | | |
| --- | --- | --- | --- | --- | --- | --- | --- | --- | --- |
| Rate (g) | Day 8 | Day 12 | Day 17 | Day 22 | Day 39 | Average | Biomass (g) | Biomass reduction (%) |  |
| 0.0 | 5.0±2.9b | 35.0±10.4a | 40.0±14.7a | 40.0±14.7a | 42.5±14.9a | 32.5±10.8a | 0.29±0.04a |  |  |
| 0.1 | 5.0±5.0b | 42.5±11.8a | 55.0±13.2a | 65.0±11.9a | 67.5±11.1a | 47.0±9.7a | 0.27±0.06a | 9.9 |  |
| 0.5 | 22.5±4.8a | 55.0±5.0a | 70.0±8.2a | 80.0±8.2a | 77.5±13.1a | 61.0±6.6a | 0.29±0.09a | 3.1 |  |
| 1.0 | 2.5±2.5b | 42.5±7.5a | 50.0±5.8a | 52.5±6.3a | 70.0±12.2a | 43.5±5.7a | 0.30±0.02a | -2.7 |  |
| P-value | 0.0168 | 0.5896 | 0.4182 | 0.1750 | 0.3880 | 0.2700 | 0.9768 |  |  |

Table 4. Continued

| Effect of water lettuce powder on germination and biomass of common ragweed | | | | | | | | | |
| --- | --- | --- | --- | --- | --- | --- | --- | --- | --- |
| Rate (g) | Day 8 | Day 12 | Day 17 | Day 22 | Day 39 | Average | Biomass (g) | Biomass reduction (%) |  |
| 0.0 | 5.0±5.0a | 30.0±10.8a | 32.5±13.1a | 32.5±13.1a | 37.5±12.5a | 27.5±10.7a | 0.17±0.07a |  |  |
| 0.1 | 0.0a | 40.0±7.1a | 45.0±9.6a | 55.0±12.6a | 57.5±13.1a | 39.5±8.2a | 0.18±0.04a | -8.2 |  |
| 0.5 | 7.5±4.8a | 47.5±7.5a | 55.0±6.5a | 50.0±11.5a | 52.5±10.3a | 42.5±7.2a | 0.33±0.06a | -95.9 |  |
| 1.0 | 12.5±2.5a | 55.0±8.7a | 55.0±8.7a | 55.0±8.7a | 55.0±8.7a | 46.5±6.8a | 0.30±0.05a | -75.3 |  |
| P-value | 0.1166 | 0.3143 | 0.4113 | 0.5409 | 0.6652 | 0.4916 | 0.2006 |  |  |

| Effect of hydrilla powder on germination and biomass of common ragweed | | | | | | | | |
| --- | --- | --- | --- | --- | --- | --- | --- | --- |
| Rate (g) | Day 8 | Day 12 | Day 17 | Day 22 | Day 39 | Average | Biomass (g) | Biomass reduction (%) |
| 0.0 | 5.0±2.9a | 35.0±6.5a | 47.5±4.8a | 40.0±7.1a | 37.5±9.5a | 33.0±2.6a | 0.15±0.04b |  |
| 0.1 | 12.5±2.5a | 47.5±7.5a | 55.0±8.7a | 55.0±8.7a | 55.0±8.7a | 45.0±6.0a | 0.26±0.04ab | -70.2 |
| 0.5 | 12.5±4.8a | 35.0±5.0a | 47.5±10.3a | 50.0±9.1a | 55.0±6.5a | 40.0±6.5a | 0.33±0.03a | -120.0 |
| 1.0 | 7.5±2.5a | 40.0a | 55.0±2.9a | 60.0a | 60.0a | 44.5±1.0a | 0.28±0.01a | -86.8 |
| P-value | 0.3949 | 0.4668 | 0.8314 | 0.3246 | 0.2019 | 0.3449 | 0.0263 |  |

Table 4. Continued

| Effect of filamentous algae powder on germination and biomass of common ragweed | | | | | | | | | |
| --- | --- | --- | --- | --- | --- | --- | --- | --- | --- |
| Rate (g) | Day 8 | Day 12 | Day 17 | Day 22 | Day 39 | Average | Biomass (g) | Biomass reduction (%) |  |
| 0.0 | 5.0±2.9a | 30.0±8.2a | 32.5±10.3a | 35.0±12.6a | 35.0±12.6a | 27.5±9.1a | 0.19±0.05a |  |  |
| 0.1 | 0.0a | 52.5±11.1a | 62.5±13.8a | 57.5±14.9a | 60.0±15.8a | 46.5±11.0a | 0.32±0.11a | -63.1 |  |
| 0.5 | 7.5±2.5a | 45.0±2.9a | 57.5±8.5a | 60.0±7.1a | 65.0±9.6a | 47.0±4.8a | 0.26±0.04a | -32.3 |  |
| 1.0 | 15.0±8.7a | 47.5±8.5a | 50.0±10.8a | 52.5±11.1a | 55.0±10.4a | 44.0±9.4a | 0.26±0.08a | -33.1 |  |
| P-value | 0.2477 | 0.3373 | 0.2726 | 0.4406 | 0.3239 | 0.3797 | 0.7038 |  |  |

| Effect of duckweed powder on germination and biomass of common ragweed | | | | | | | | | |
| --- | --- | --- | --- | --- | --- | --- | --- | --- | --- |
| Rate (g) | Day 8 | Day 12 | Day 17 | Day 22 | Day 39 | Average | Biomass (g) | Biomass reduction  (%) |  |
| 0.0 | 5.0±2.9a | 32.5±2.5a | 35.0±5.0a | 37.5±4.8a | 40.0±5.8a | 30.0±3.8 a | 0.20±0.02a |  |  |
| 0.1 | 7.5±4.8a | 30.0±5.8a | 32.5±7.5a | 45.0±9.6a | 52.5±7.5a | 33.5±6.8a | 0.24±0.03a | -17.4 |  |
| 0.5 | 17.5±8.5a | 45.0±10.4a | 45.0±10.4a | 52.5±11.8a | 60.0±12.2a | 44.0±10.1a | 0.23±0.06a | -12.4 |  |
| 1.0 | 12.5±2.5a | 42.5±7.5a | 47.5±7.5a | 47.5±7.5a | 52.5±7.5a | 40.5±6.1a | 0.26±0.07a | -30.4 |  |
| P-value | 0.4539 | 0.3818 | 0.5018 | 0.7438 | 0.5157 | 0.5516 | 0.7242 |  |  |

Table 5. Effect of aquatic weed extracts on germination rate of amaranth.

|  | Germination rate (%) | |
| --- | --- | --- |
|  | Dilution (v/v) | |
| Treatment | 1/100 | 1/10 |
| Water control | 96.3 ± 2.4a | 100.0 a |
| Aqueous ethanol control | 97.5 ± 2.5a | 0.0 c |
| Muskgrass | 95.0 ± 2.9a | 0.0 c |
| Water hyacinth | 87.5 ± 9.5ab | 0.0 c |
| Water lettuce | 85.0 ± 8.7ab | 0.0 c |
| Hydrilla | 85.0 ± 6.5ab | 0.0 c |
| Filamentous algae | 70.0 ± 12.9b | 0.0 c |
| Duckweed | 100.0 a | 0.0 c |

Table 6. Effect of aquatic weed extracts on germination rate of common ragweed.

|  | Germination rate (%) | | |
| --- | --- | --- | --- |
|  | Dilution (v/v) | | |
| Treatment | 1/100 | | 1/10 |
| Water control | 72.5 ± 4.8ab | | 82.5 ± 4.8ab |
| Aqueous ethanol control | 67.5 ± 4.8abc | 25.0 ± 12.6de | |
| Muskgrass | 65.0 ± 2.9abc | 25.0 ± 8.7de | |
| Water hyacinth | 65.0 ± 8.7abc | 32.5 ± 2.5cde | |
| Water lettuce | 65.0 ± 6.5abc | 37.5 ± 10.3bcde | |
| Hydrilla | 65.0 ± 5.0abc | 35.0 ± 12.6bcde | |
| Filamentous algae | 72.5 ± 4.8ab | 20.0 ± 8.2e | |
| Duckweed | 60.0 ± 9.1ab | 20.0 ± 4.1e | |

Model 1. **PROC GLIMMIX program for analysis of fall armyworm RGR data**

**proc** **glimmix** data=data;

title2'Fall Armyworm RGR';

class Assay Day Trt ;

model RGR = Trt / ddfm=kr htype=**3**;

random Assay Day(Assay);

lsmeans Trt / diff adjust=tukey lines;

**run**;

Model 2. **PROC GLIMMIX program for analysis of leaf area data**

**proc** **glimmix** data=data;

title2'Leaf Area';

class Assay Plate Trt Time;

model Area = Trt Time Trt*Time/ htype=**3** ddfm=kr;

random Assay Plate(Assay) Trt*Plate(Assay);

lsmeans Trt Time / diff adjust=tukey lines;

lsmeans Trt*Time / slice=Time;

lsmeans Trt*Time / slicediff=Time adjust=tukey;

**run**;
